# Structural Characterization and In Vitro and In Silico Studies on the Anti-*α*-Glucosidase Activity of Anacardic Acids from *Anacardium occidentale*

**DOI:** 10.3390/foods13244107

**Published:** 2024-12-19

**Authors:** Ana Priscila Monteiro da Silva, Gisele Silvestre da Silva, Francisco Oiram Filho, Maria Francilene Souza Silva, Guilherme Julião Zocolo, Edy Sousa de Brito

**Affiliations:** 1 Embrapa Agroindústria Tropical, Fortaleza 60511-110, CE, Brazil; priscilamonteiro62@hotmail.com (A.P.M.d.S.); gihchemistry@gmail.com (G.S.d.S.); oiramfilho@yahoo.com.br (F.O.F.); guilherme.zocolo@embrapa.br (G.J.Z.); 2 Department of Chemical Engineering, UFC, Federal University of Ceará, Campus do Pici, Bloco 709, Fortaleza 60455-760, CE, Brazil; 3 Research and Development of Medicines, Federal University of Ceará, Rua Coronel Nunes de Melo 1000, Rodolfo Teófilo, Fortaleza 60420-275, CE, Brazil; lenolysilva@hotmail.com; 4 Embrapa Soja, Londrina 86085-981, PR, Brazil; 5 Embrapa Alimentos e Territórios, Maceió 57020-050, AL, Brazil

**Keywords:** antidiabetic compounds, anacardic acid, alkyl phenol, *α*-glucosidase inhibition, molecular docking, pharmacokinetic properties, drug likeness, by-product

## Abstract

The growing focus on sustainable use of natural resources has brought attention to cashew nut shell liquid (CNSL), a by-product rich in anacardic acids (AAs) with potential applications in diabetes treatment. In this study, three different AAs from CNSL, monoene (15:1, AAn1), diene (15:2, AAn2), and triene (15:3, AAn3), and a mixture of the three (mix) were evaluated as *α*-glucosidase inhibitors. The samples were characterized by combining 1D and 2D NMR spectroscopy, along with ESI-MS. In vitro assays revealed that AAn1 had the strongest inhibitory effect (IC_50_ = 1.78 ± 0.08 μg mL^−1^), followed by AAn2 (1.99 ± 0.76 μg mL^−1^), AAn3 (3.31 ± 0.03 μg mL^−1^), and the mixture (3.72 ± 2.11 μg mL^−1^). All AAs significantly outperformed acarbose (IC_50_ = 169.3 μg mL^−1^). In silico docking suggested that polar groups on the aromatic ring are key for enzyme–ligand binding. The double bond at C15, while not essential, enhanced the inhibitory effects. Toxicity predictions classified AAs as category IV, and pharmacokinetic analysis suggested moderately favorable drug-like properties. These findings highlight AAs as a promising option in the search for new hypoglycemic compounds.

## 1. Introduction

Cashew nut shell liquid (CNSL) is a by-product of cashew processing, accounting for approximately 30–35% of the total weight of the shells [1]. The large volume of CNSL produced presents environmental challenges, especially in light of increasing global cashew nut production [2]. To address these issues, there is growing interest in repurposing CNSL as a valuable raw material for producing functional products in sectors such as medicine, nutraceuticals, and biodegradable materials [2,3,4,5,6,7,8,9]. In addition to the environmental advantage, CNSL as a source of bioactive compounds, being an industrial by-product and a non-edible material, offers advantages over products currently explored for medicinal chemistry activities (natural and fossil-based chemicals) from an economic and ethical point of view [10].

The composition of CNSL changes according to the extraction process employed, resulting in either natural or technical versions [11]. CNSL is recognized for its rich content of phenolic lipids, which include bioactive compounds, such as anacardic acids (AAs), cardols, and cardanols [7,12]. Among these, AAs have garnered considerable attention because of their impressive therapeutic properties [5,13], making them promising candidates for healthcare applications. Natural CNSL extracts can contain anacardic acid concentrations as high as 60–70% [14], highlighting the importance of studying AAs derived from CNSL for advancements across various fields.

In cancer research, anacardic acids have demonstrated potent inhibitory effects on histone acetyltransferase [15] and have shown antiproliferative and cytotoxic effects [16]. They also interfere with the interactions of anti-apoptotic and pro-apoptotic proteins [17]. Additionally, AAs have been investigated for their anxiolytic [18] and neuroprotective [19,20] properties, as well as their anti-angiotensin type 1 [21], anti-metalloproteinase [22], anti-*α*-glucosidase [23,24], and anti-acetylcholinesterase activities [25,26], as well as their potential against viral infections, such as SARS-CoV-2 [27] and hepatitis C [28]. They exhibit antifungal [29] and antibacterial properties [29,30], as well as noteworthy larvicidal potential [31].

The study of AAs is emerging as a significant trend in the research of treatments for metabolic syndrome, which is related to a greater likelihood of developing type 2 diabetes and heart diseases [13]. As metabolic syndrome becomes more prevalent globally, the demand for effective natural therapeutic agents is growing. AAs can effectively reduce liver fat accumulation and improve glucose tolerance in mice subjected to diets high in fat and sugar [15], suggesting a role for AAs in addressing insulin resistance and other metabolic dysfunctions. Additionally, the antioxidant properties of AAs [25,32,33] demonstrate their ability to protect against oxidative stress, which is associated with metabolic disorders, including diabetes [15,34,35].

Inhibiting the α-glucosidase enzyme may help control postprandial blood glucose spikes. Recent studies conducted by Hu et al. [23] and Jiang et al. [24] described a notable *α*-glucosidase inhibitory effect in anacardic acid derivatives extracted from *S. samarangense* and *S. jambos*. However, the inhibitory effects of anacardic acids from CNSL remain underexplored, presenting a critical gap that requires further investigation. This study aims to assess the capacity of anacardic acids from CNSL to inhibit α-glucosidase, provide MS and NMR structural elucidation of these congeners, and conduct ADMET (absorption, distribution, metabolism, excretion, and toxicity) studies to evaluate their pharmacokinetic properties. By utilizing CNSL, we aim to promote sustainable resource use, minimize waste in the cashew industry, and contribute to solutions for rising health issues.

## 2. Materials and Methods

### 2.1. Materials and Chemicals

In this study, anacardic acids were isolated from natural CNSL extracted from cashew nut shells by a pressing process, as described by Oiram Filho et al. [12]. The nuts were obtained from cashews from the experimental field of Embrapa Agroindústria Tropical in Pacajus-CE, Brazil. α-Glucosidase (α-Glu) from *Saccharomyces cervisiae* (3. 2. 1. 20), acarbose, dimethyl sulfoxide (DMSO), glutathione, *p*-nitrophenyl-*α*-*D*-glucopyranoside (pNPG), and DMSO-*d*_6_ were purchased from Sigma-Aldrich (St. Louis, MO, USA). Buffer reagents and Na_2_CO_3_ were purchased from Vetec (Verden, Germany).

### 2.2. Anacardic Acid Isolation via Preparative HPLC

The analytical chromatographic method previously developed by Oiram Filho et al. [36] served as a foundation for scaling up to the preparative scale. A preparative chromatographic system was utilized to obtain an extract containing a mixture of three anacardic acids (mix) as well as to purify each compound: anacardic acid monoene (AAn1), diene (AAn2), and triene (AAn3).

A preparative HPLC apparatus that included a quaternary gradient module (Waters 2555, Milford, CT, USA), a UV-Vis detector (Waters 2489), and a fraction collector (Waters Fraction Collector III, Dublin, Leinster, Ireland) was utilized. The instrument was equipped with a C18 reversed-phase column (100 × 19 mm, 5 µm, Waters SunFire Prep). For the fraction containing the mixture of anacardic acids, the peaks corresponding to the three anacardic acids were collected into a single fraction. In contrast, the purified compounds (AAn1, AAn2, and AAn3) were collected individually, peak by peak, according to their respective retention times, as described by Oiram Filho et al. [12]. The chromatographic conditions employed included a mobile phase formulated with methanol and water in isocratic mode (90:10), both of which were acidified with acetic acid (1%). The flow rate was set at 10 mL min^−1^ for 40 min at 25 °C, with a fixed injection volume of 1 mL and an injection load of 100 mg of cashew nut shell liquid [37].

### 2.3. Structural Confirmation of Anacardic Acids by UPLC-QTOF-MS^E^

In order to obtain the identification of the three AAs, (15:3), (15:2), and (15:1), performed with accuracy in the HPLC-DAD system, a high-resolution analysis was performed on a Waters Acquity UPLC analyzer coupled to a Xevo Quadrupole and Time-of-Flight mass system (QTOF, Water, Milford, MA, USA), equipped with an electrospray interface (ESI) to perform the unambiguous identification of the three different anacardic acids present in CNSL. Separations were realized in the C_18_ column (Waters Acquity UPLC C_18_, 150 mm × 2.1 mm, 1.7 µm). The profile of the metabolites contained in the sample was initially obtained by an exploratory gradient, with the mobile phase being composed by H_2_O (A) and acetonitrile (B), each of these containing formic acid (0.1% *v*/*v*). The samples were subjected to the following exploratory gradient: 2–95% of solvent (B) in 20 min at a flow rate of 500 µL.min^−1^. The analysis was conducted in negative ionization mode in the range of 100–1200 Da. The ESI source conditions were defined as follows: capillary voltage 2800 V, cone voltage 40 V, source temperature 120 °C, solvation temperature 330 °C, cone gas flow 20 L.h^−1^, gas flow of solvation 600 L.h^−1^, and MCP (microchannel plate voltage) detector at 1900 V. The structural identification of the metabolites present in the sample was carried out using the molecular formulas and *m*/*z* values obtained from high-resolution spectra, observed at the highest-intensity chromatographic peaks through MassLynx 4.1 software (Water Corporation). The structural proposals of the AnAc molecules were performed using the MS/MS data, over the establishment of rational fragmentation patterns [38].

### 2.4. 1D and 2D NMR Spectroscopy

The spectra of proton nuclear magnetic resonance (^1^H NMR) for all compounds were measured at 600 MHz, while the carbon-13 (^13^C NMR) spectra were recorded at 150 MHz. Approximately 18 mg of each material (AAn1, AAn2, AAn3, or their mixture) was directly solubilized in 550 µL of DMSO-*d*_6_. The solution was mixed thoroughly, filtered, and then placed into 5 mm NMR tubes. NMR experiments were conducted using an Agilent 600 MHz spectrometer (Palo Alto, CA, USA) that featured a 5 mm inverse detection One Probe™. For the acquisition of ^1^H NMR spectra, a pre-saturation pulse sequence was utilized to effectively suppress the water signal at *δ*_H_ 4.75 ppm. To ensure the comprehensive identification of anacardic acids, additional 2D NMR analyses, including homonuclear correlation spectroscopy (^1^H-^1^H, COSY), heteronuclear single quantum coherence (^1^H-^13^C, HSQC), and heteronuclear multiple-bond correlation (^1^H-^13^C, HMBC), were conducted. The 2D NMR studies were conducted via previously reported standard spectrometer pulse sequences [39,40]. More details on the NMR parameters can be found in the Supplementary Materials. The spectra were analyzed and processed with the help of Mnova software (v12.0, Mestrelab Research). Chemical shifts are expressed in parts per million (ppm, *δ*) with reference to DMSO-*d_6_*. The residual solvent signals were used as reference values, with *δ_H_* set at 2.50 ppm for protons and *δ*_C_ at 39.51 ppm for carbons. In the 1D NMR spectra, the resolution was enhanced by applying Gaussian–Lorentzian window functions (with GB set to 1 and line broadening to 0.3). Baseline correction was managed via a fifth-order polynomial, whereas phase correction was executed manually. For the ^1^H-^13^C HSQC experiments, a 90°-shifted sine window function was employed to optimize the resolution, complemented by appropriate zero-filling and linear prediction, with a third-order polynomial applied for baseline correction. The multiplicity-edited ^1^H–^13^C HSQC pulse sequences produced inverted signs between the peaks of =CH_2_/–CH_2_– groups and –CH–/–CH_3_ groups. For the ^1^H-^13^CHMBC and ^1^H-^1^HCOSY experiments, the line resolution was enhanced by applying a sine (0) window function, zero-filling, and linear prediction. Post-processing steps included baseline correction with a third-order polynomial function and the omission of phase correction.

### 2.5. α-GLU Inhibitory Assay

The in vitro *α*-GLU (*α*-glucosidase) inhibition assay was conducted via an adapted methodology [41,42,43]. Initially, dimethyl sulfoxide (DMSO) was used to dissolve the samples (mix, AAn1, AAn2, and AAn3) at concentrations of 2, 1, 0.5, 0.25, 0.125, and 0.0625 mg mL^−1^. A volume of 3 µL of each solution was pipetted into a 96-well microplate, then 47 µL of assay buffer (100 mM sodium phosphate, pH 6.8) was added, resulting in 6.4% DMSO in the buffer. Subsequently, 50 µL of glutathione solution (2 mg mL^−1^) and 50 µL of *α*-glucosidase solution (0.4 U mL^−1^) were added. The mixture was shaken and incubated for 5 min at 25 °C. The reaction started when 50 µL of pNPG (5 mM) was added, and after 10 min, 50 µL of Na_2_CO_3_ (0.1 M) was added to stop the reaction. A negative control was prepared using DMSO (3 µL) instead of the sample, while acarbose was the positive control of the study. The absorbance of *p*-nitrophenol was measured at 415 nm via a microplate reader (iMark, Bio-Rad, Hercules, CA, USA). The percentage of inhibition (% inhibition) was calculated as follows:

(1) $$\%\;inhibition=\left[1-\frac{A_{sample}}{A_{control}}\right]\times 100$$

where $A_{sample}$ represents the absorbance of the sample and $A_{control}$ represents the absorbance of the negative control (absence of inhibitor).

### 2.6. Statistical Analysis

Statistical data were obtained with GraphPad Prism Software Version 8.0 (GraphPad Software, San Diego, CA, USA). The inhibition percentages from two separate experiments (N = 2), executed in triplicate (n = 3), were examined using two-way ANOVA and subsequently assessed with Tukey’s multiple comparisons test. Results are expressed as means ± SDs, with IC_50_ values calculated using non-linear regression analysis. A *p*-value < 0.05 was interpreted as indicative of statistical significance.

### 2.7. Molecular Docking

First, the analogous acids were sketched in Marvin V 24.1, ChemAxon (https://chemaxon.com/ (accessed on 6 February of 2024)), and then the ideal conformations of the compounds were calculated via the semiempirical method PM6 in the Gaussian 09 program [44]. The *Saccharomyces cerevisiae* S288C *α*-glucosidase (PDB ID: 4J5T) structure was downloaded from the Protein Data Bank (Resolution: 2.04 Å). The protonation states of the amino acid residues of the enzyme were calculated at physiological pH via the PROPKA module of the PDB2PQR server (https://server.poissonboltzmann.org/pdb2pqr (accessed on 11 march of 2024)). Molecular docking calculations were performed via AutoDock Vina 1.1 software [45] and AutoDock tools [46]. The binding sites were set to a box size of −10.27 Å × −28.53 Å × 2.63 Å. The receptor grid box was constructed with dimensions of 29 Å in each direction (x, y, and z), centered on the respective ligands to ensure that the binding pocket could adequately accommodate any ligand. All the bonds in the ligands (-*E*/*Z* stereoisomers) were designated as nonrotatable. Additionally, the validation of the docking methodology was conducted through a re-docking process, following a procedure akin to that outlined by Ha et al. [47]. This process involved assessing the docking outcomes of the most stable conformations of various anacardic ligands, alongside an antidiabetic drug utilized as a positive control. The 2D and 3D protein–ligand interactions were analyzed via the ProteinsPlus web service (https://proteins.plus (accessed on18 of march of 2024)) and the PyMOL Molecular Graphics System, Version 3.0 Schrödinger, LLC (https://www.pymol.org/pymol.html (accessed on 19 march of 2024)). A root-mean-square deviation (RMSD) value of 0.0 Å was used as a criterion to identify the most accurate predictions for ligand-binding poses. Generally, an RMSD value lower than 2 Å indicates a successful binding mode prediction [48]. Therefore, a RMSD of 0.0 Å signifies a high level of accuracy and reliability in the predicted binding conformations. The findings are presented as the free binding energy of the receptor–ligand complex.

### 2.8. ADMET and Drug-Likeness Analysis

The prediction of anacardic acids’ toxicity was performed via the website ProTox-II (https://tox-new.charite.de/protox_II/ (accessed on 25 march of 2024)). Toxicity classification was based on the standards [49] of the globally harmonized system of classification and labeling of chemicals (https://www.osha.gov/hazcom (accessed on 26 march of 2024)). Pharmacokinetic (ADME) and drug-likeness profiles (Lipinsky, Ghose, Veber, Egan, and Muegge models) were evaluated in the pkCSM (http://biosig.unimelb.edu.au/pkcsm/ (accessed on 27 march of 2024)) and SwissADME webservers (http://www.swissadme.ch (accessed on 28 march of 2024)), respectively.

## 3. Results and Discussion

### 3.1. Structural Analysis of CNSL Anacardic Acids

Anacardic acids, also known as 6-alkylsalicylic acids [50], exhibit a structural composition that includes a trisubstituted benzene ring that features a hydroxyl group, a carboxyl group, and an alkyl chain, which can be either saturated or unsaturated. The arrangement of these functional groups on the ring significantly influences the biological, chemical, and physical characteristics of the compounds [6,51].

Three anacardic acids were isolated and identified from CNSL, which appeared as yellow oils: monoene AAn1 (15:1), diene AAn2 (15:2), and triene AAn3 (15:3). In the analysis of AAn1, AAn2, and AAn3 using negative-ion electrospray ionization mass spectrometry (ESI-MS), the corresponding precursor ions, [M-H]^−^, were identified at *m*/*z* 345.2397, 343.2243, and 297.2213, respectively [12,37,52]. All three compounds exhibited characteristic fragmentation, resulting in the loss of 44 Da, corresponding to one CO_2_ molecule, which produced fragment ions at *m*/*z* 301.2517, 299.2364, and 297.2213. The ESI-MS spectra of anacardic acids can be found in Figures S1–S3 in the Supplementary Materials.

To gain deeper insights into the structure of compound AAn1, we conducted a detailed analysis via ^1^H and ^13^C NMR spectroscopy, along with findings from the ^1^H–^13^C HSQC, ^1^H-^1^H COSY, and ^1^H-^13^C HMBC contour maps, which revealed several distinct resonance signals typical of a trisubstituted benzene structure.

**Figure 1:**
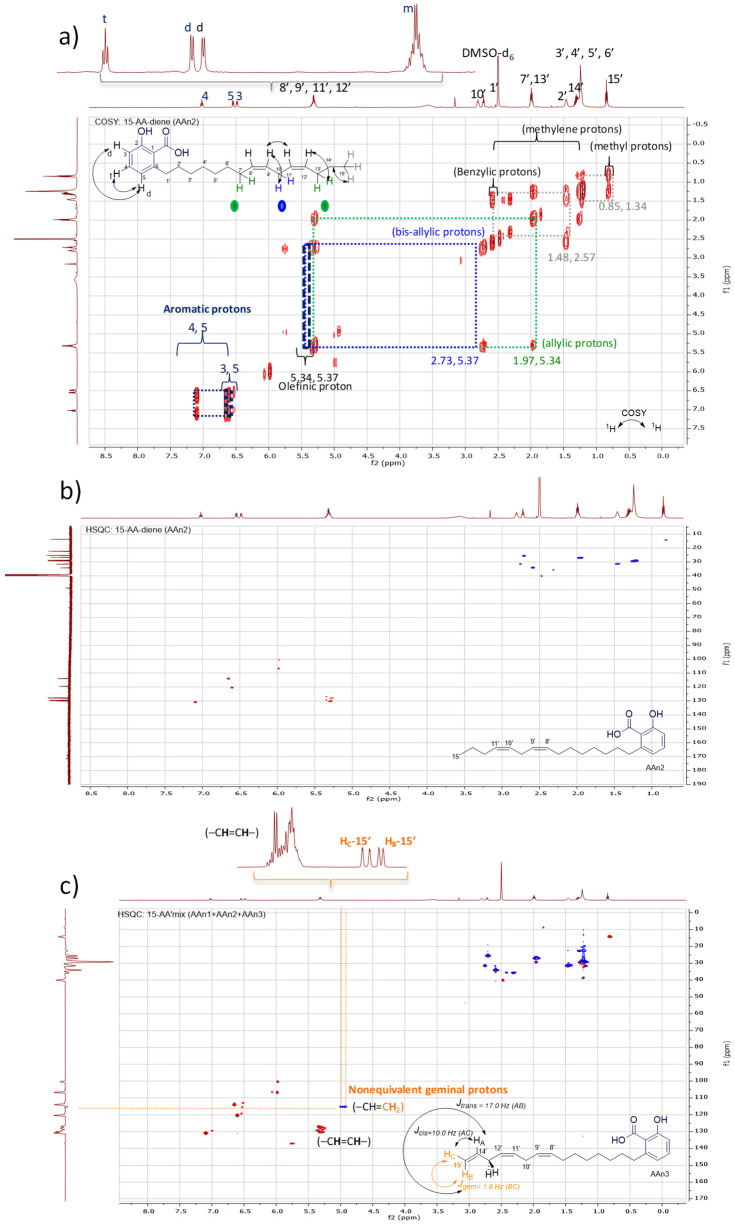
Analysis of anacardic acids via NMR: (**a**) 2D ^1^H-^1^H COSY contour map of AAn2, (**b**) 2D ^1^H-^13^C HSQC contour map of AAn2, and (**c**) 2D ^1^H-^13^C HSQC contour map of the anacardic acid mixture (mix: AAn1 + AAn2 + AAn3), presents the antiphase relationship between the =C**H**_2_ and –C**H**_2_– groups (blue) and the -C**H**-/-C**H**_3_ groups (red).

The ^1^H NMR spectra of all anacardic acids revealed the presence of three aromatic protons in the benzene ring, which were detected at *δ*_H_ 7.02 (triplet, *J* = 7.7 Hz—H-4), *δ*_H_ 6.54 (doublet, *J* = 7.7 Hz—H-5), and *δ*_H_ 6.49 (doublet, *J* = 7.7 Hz—H-3). This ortho-coupling pattern is characteristic of trisubstituted benzenes. The correlations between H-4 and H-5, as well as those between H-5 and H-3, were corroborated through analysis of the ^1^H-^1^H COSY contour map (Figure 1a), which confirmed the presence of the three-spin system. Additionally, the detection of the CO_2_H group was further supported by its peak at *δ*_C_ 173.7 in the ^13^C NMR spectrum.

Furthermore, a triplet signal (*J* = 7.8 Hz) was observed for the benzylic methylene at *δ*_H_ 2.57 ppm, which was correlated with the carbon resonance at *δ*_C_ 33.1 (C-1′). This proton signal also exhibited cross-peaks in the ^1^H-^13^C HMBC spectrum with C-5 and C-6, providing additional structural information. Thus, it was confirmed that the aliphatic chain is attached to the aromatic ring at position C-6, aligning with the structural characteristics of 6-alkylsalicylic acid.

The observed set of resonance signals in the ^1^H NMR spectrum, along with the cross-peaks in the ^1^H-^1^H COSY and ^1^H-^13^C HMBC contour maps, combined with the multiplicity data and coupling constants, clearly supported the presence of a typical spin-coupling system associated with (1,2,3)-trisubstituted benzene rings, which are commonly referred to as (1,2,6)-trisubstituted benzenes [23,53]. For the purposes of this study, we adopted the (1,2,6) numbering convention for the AA compounds.

Notably, the shifts in the chemical environment of the aromatic hydrogen and carbon atoms for all anacardic acids were not affected by variations in the number and position of unsaturation, as previously reported [54]. Similarly, this characteristic was also observed in the olefinic protons, as shown in Figure 1. The hydrogen atoms directly bonded to sp^2^-hybridized carbons (C-8′/9′ and C-11′/12′) in all compounds (AAn1 + AAn2 + AAn3) exhibited typical signals at approximately *δ*_H_ 5.31 ppm. In contrast, an alkyl chain’s terminal bond with two geminal protons was detected at approximately *δ_H_* 4.99 ppm, which corresponded to the protons directly bonded to C-15′ of the triene. The terminal olefinic cross-peaks in blue (=C**H**_2_; C-15′ and H-15′) of the triene AAn3 are highlighted in the ^1^H-^13^C HSQC contour map in Figure 1c, demonstrating their antiphase relationship with the other sp^2^ carbon signals (–C**H**=C**H**–) in the same region, which are shown in red.

Although the overlapping olefinic proton signals in the hydrogen NMR spectrum made determining the geometry of the double bonds challenging, the ^1^H-^1^H COSY analysis offered clear correlations for signal assignments. These assignments were further corroborated by the relationships identified in the ^1^H-^13^C HMBC data.

The remaining hydrogen atoms of the C15 alkenyl side chain in the AA congeners were assigned in decreasing order, as follows: bisallylic (=CH–C**H**_2_–CH=; H-10′ and H-13′, considering the numbering and assignment of each AA proton signal in Table 1) observed at *δ*_H_ 2.73 ppm > benzylic methylene (C_6_H_5_-C**H**_2_; H-1′) at *δ*_H_ 2.57 ppm > allylic (C**H**_2_-CH=CH–; H-7′, H-10′, and H-13′) at *δ*_H_ 1.97 ppm. In addition, a homobenzylic methylene signal was observed at *δ*_H_ 1.48 ppm (H-2′). The remaining methylene (–CH_2_–) hydrogens (H-3′ through H-6′ and H-11′ through H-14′) appeared as more intense overlapping signals at around *δ*_H_ 1.21 to 1.26 ppm.

Furthermore, the terminal methyl group of the alkenyl side chain (H-15′) was observed at *δ*_H_ 0.85 ppm in AAn1 and AAn2. In contrast, AAn3 displayed an additional terminal olefinic group positioned at the chain’s end (–CH_2_–CH=C**H**_2_; Figure 1c), where nonequivalent protons were observed at *δ*_H_ 4.99 ppm (dd, *J* = 17.0 and 10.0 Hz for H-14′, reflecting *trans* and *cis* coupling) and *δ*_H_ 5.01 ppm (dd, *J* = 10.0 and 1.9 Hz for H-15′, indicating *cis* and *geminal* coupling).

The complete assignments of the proton and carbon signals for the monoene, diene, and triene AAs are detailed in Table 1, in accordance with the MS and NMR data reported in the literature [5,21,23,58]. As a result, the structures of AAn1, AAn2, and AAn3 were determined to be 2-hydroxy-6-(pentadec-8-en-1-yl) benzoic acid, 2-hydroxy-6-(pentadeca-8,11-dien-1-yl) benzoic acid, and 2-hydroxy-6-(pentadeca-8,11,14-trien-1-yl)benzoic acid, respectively. Additionally, chromatograms of the mixed sample (mix = AAn1 + AAn2 + AAn3), negative-ion ESI-MS spectra of anacardic acids, and both one-dimensional and two-dimensional NMR spectra of representative samples are available in the Supplementary Materials.

### 3.2. In Vitro Anti-α-Glucosidase Activity

**Figure 2:**
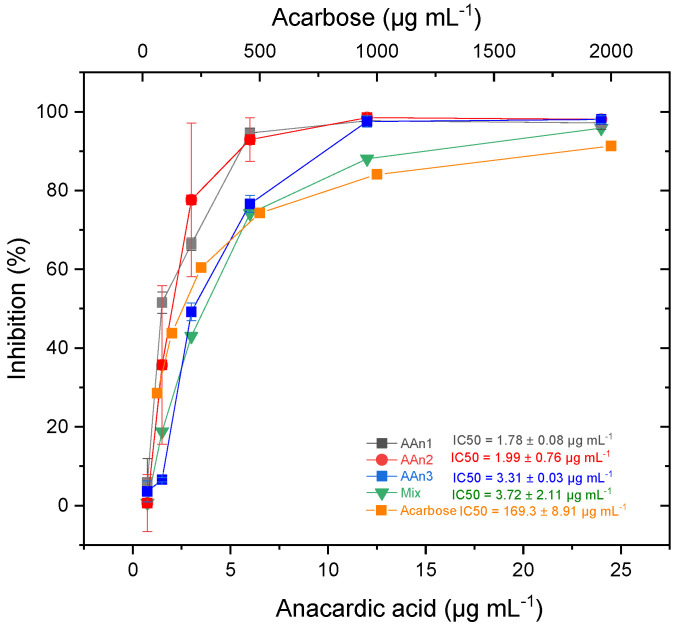
Inhibition *of α*-GLU by anacardic acids (0.75 to 24 µg mL^−1^) compared with acarbose (62.5 to 2000 µg mL^−1^). The values are presented as the means ± SDs.

An in vitro enzymatic experiment was performed to evaluate the inhibitory activity of anacardic acids against the *α*-glucosidase enzyme (*α*-GLU). Inhibition of this enzyme is one of the therapeutic approaches used to treat type 2 diabetes [59,60]. Figure 2 shows the dose-dependent impact of anacardic acids and acarbose on the inhibition of *α*-GLU activity, along with the IC_50_ values for each sample.

All the samples demonstrated a profile in which enzyme inhibition increased with the increasing concentration of the tested compounds. The monoene AAn1 had the greatest inhibitory effect, presenting an IC_50_ value of 1.78 ± 0.08 μg mL^−1^ (5.1 ± 0.2 μM), followed by the diene AAn2 with IC_50_ = 1.99 ± 0.76 μg mL^−1^ (5.8 ± 2.2 μM), the triene AAn3 with IC_50_ = 3.31 ± 0.03 µg mL^−1^ (9.7 ± 0.1 μM), and the mixture of anacardic acids (mixed sample, IC_50_ = 3.72 ± 2.11 µg mL^−1^). These findings highlighted the superior *α*-glucosidase inhibitory effectiveness of anacardic acids compared with that of acarbose with IC_50_ = 169.3 ± 8.91 µg mL^−1^ (262.2 ± 13.8 μM). These results aligned with the data obtained by Hu et al. [23], who isolated derivatives of anacardic acid (2-[(*Z*)-nonadec-14-enyl]-6-hydroxybenzoic acid) from the leaves of *Syzygium samarangense*. These compounds significantly inhibited *α*-glucosidase. The range of IC_50_ values reported by the authors varied from 0.75 to 3.60 μM.

Tukey multiple comparisons tests were performed to evaluate the differences in the mean percentage inhibition among the anacardic acid samples (AAn1, AAn2, and AAn3) and the mixture (mix). The results are presented with the mean differences, 95% confidence intervals (CIs) for the differences, statistical significance, and adjusted *p*-values in Table S1 (Supplementary Materials). Statistically, there was no significant difference between the inhibitory effects of AAn1 and AAn2 or between AAn3 and the mixture (*p* > 0.05). The most significant comparisons were observed between the anacardic acids AAn1 (*p* < 0.0001) and AAn2 (*p* < 0.05), with the mixture of anacardic acids demonstrating the most pronounced inhibitory effect.

### 3.3. Molecular Docking Studies

In Section 3.1, the structural characteristics of anacardic acids were elucidated through MS and NMR data, revealing that they feature a 1,2,6-trisubstituted benzene ring with substituents, including a hydroxyl group, a carboxylic acid, and an alk(en)yl chain containing 15 carbon atoms. However, the connection between the scaffold of anacardic acids and their anti-*α*-glucosidase activity is not yet fully understood. As previously reported [22], the variability in these structural characteristics leads to a combination of polar and nonpolar functional groups, resulting in distinct physical and chemical properties, as well as different biological activities, such as the inhibition of *α*-glucosidase.

Docking calculations have proven to be especially effective in pinpointing the molecular targets of nutraceuticals for the management of diseases [61]. Protein-ligand docking is a vital method in computational drug development, allowing researchers to predict atomic-level interactions between small molecules and proteins [62]. One key parameter derived from these docking studies is the predicted binding affinity, which is a crucial determinant of how the structure of amino acids (AAs) affects their interaction with the target protein [63]. A more negative binding affinity signifies a stronger interaction between the enzyme and the ligand, resulting in heightened stability of the enzyme–ligand complex [64].

For the purpose of exploring the interactions between anacardic acids and the enzyme α-glucosidase (α-GLU, PDB 4J5T), in silico molecular docking simulations were performed, focusing on the enzyme’s active site [65]. The primary aim was to predict the modes of interaction and binding energies for several anacardic acids, focusing on three specific compounds: monoene AAn1, diene AAn2, and triene AAn3, in comparison to acarbose. Additionally, we evaluated a specific derivative, referred to as the 15-carbon anacardic acid analog (designated AAn0). This compound was included in the analysis to evaluate the impact of the absence of a double bond in its structure, in comparison to the other anacardic acids. The predicted binding affinity values (measured in kcal mol^−1^) for a series of AAs derived from the cashew nut shell of *A. occidentale* are shown in Table 2.

**Figure 3:**
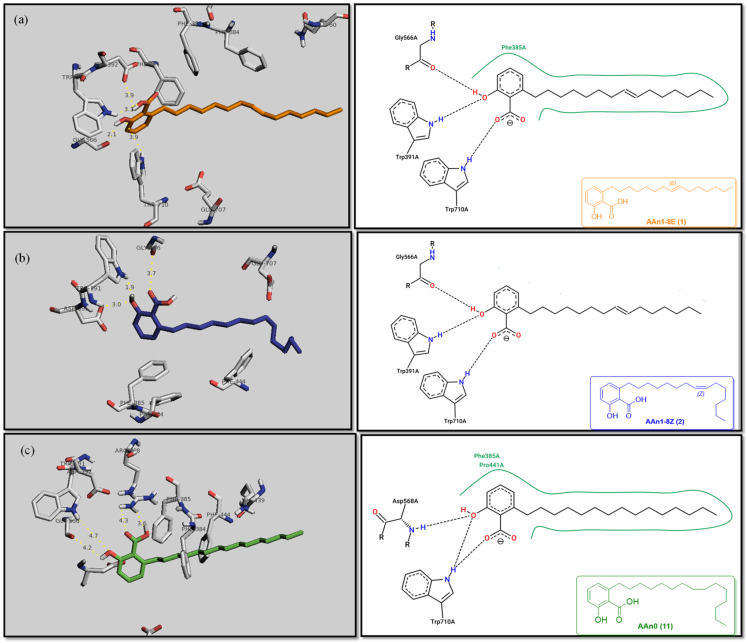
3D and 2D binding modes of anacardic acids in the active site of *α*-glucosidase: AAn1-8*E* (**a**), AAn1-8*Z* (**b**), and AAn0 (**c**). Atom colors: oxygen (O: red), nitrogen (N: blue), hydrogen (H: white), and carbon from AAn1 (gray), AAn2 (orange), and AAn0 (green). In the 2D representation, only the most favorable hydrogen bonds (distances up to 3.7 Å) are shown, whereas the 3D representation includes all hydrogen bonds (distances up to 4.7 Å).

**Figure 4:**
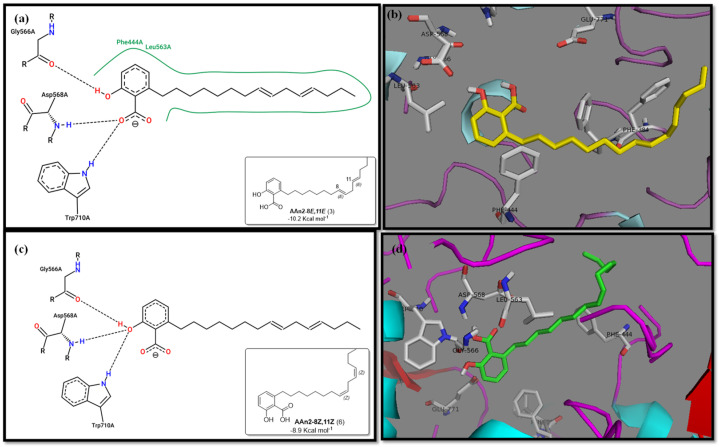
2D and 3D representations of the binding modes of AAn2-8*E*,11*E* (n = 2, diene, −10.2 kcal mol^−1^) and AAn2-8*Z*,11*Z* (n = 2, diene, 8.9 kcal mol^−1^) (**a**,**c**) at the active site of *α*-glucosidase (**b**,**d**).

**Figure 5:**
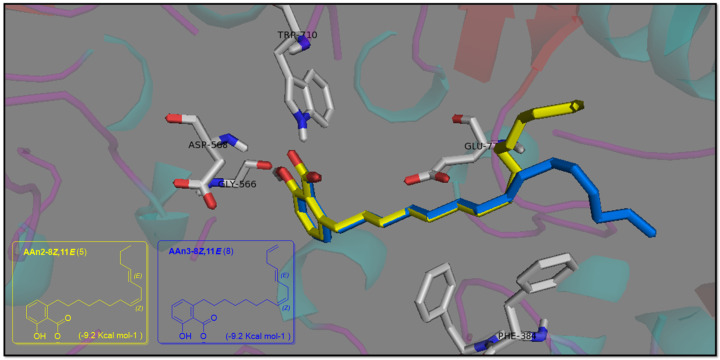
3D representation of the superimposed AAn2-8*Z*, 11*E* (n = 2, dieno, −9.2 kcal mol^−1^) and AAn3-8*Z*, 11*E* (n = 3, triene, −9.2 kcal mol^−1^) ligands docked at the catalytic site of *α*-glucosidase.

The results of the molecular docking study supported the experimental data on enzyme inhibition presented in Section 3.2, demonstrating that all anacardic acids exhibited significant inhibitory potential. More specifically, the docking study revealed that AAn1 (−8.9 to −9.9 kcal mol^−1^), AAn2 (−8.9 to −10.2 kcal mol^−1^), and AAn3 (−8.4 to −9.2 kcal mol^−1^) presented binding affinities equal to or lower than that of acarbose (−8.4 kcal mol^−1^) within the catalytic site of the *α*-glucosidase enzyme. Figures S5 and S6 provide 2D and 3D representations, respectively, of acarbose docked in its optimal conformation within the *α*-glucosidase enzyme–ligand complex. Anacardic acids (AAn0, AAn1, AAn2, and AAn3) interacted with the active site of *α*-GLU primarily through a combination of hydrogen bonds and hydrophobic forces, akin to the interactions observed with the pharmaceutical agent acarbose [47,64,65,66]. Furthermore, hydrogen bond interactions with glycine (GLY566; Figure 3, Figure 4 and Figure 5) were observed in all the studied anacardic acids (except AAn0; see Table 2), indicating that this residue is essential for the formation of the enzyme–ligand complex. Notably, this interaction has also been observed with acarbose [65,67].

Acarbose, used as a reference standard in experimental assays, is an oligosaccharide that is clinically used to slow carbohydrate digestion, thereby reducing blood glucose levels after meals in individuals with type 2 diabetes [68]. Critical catalytic residues of *α*-GLU, including ASP568, ARG387, PHE384, LYS439, GLY566, GLY383, and TRP391 [64,69,70], are shown in Figures S5–S7.

The validation of the docking method was achieved through a re-docking procedure, as described by Ha et al. [47]. This procedure involved the evaluation of the best docking poses of the most stable conformations of various ligands within the active site of the crystal structure of α-glucosidase (PDB ID: 4J5T). This evaluation was deemed necessary because acarbose, a known inhibitor, is not co-crystallized in the three-dimensional structure of the 4J5T enzyme.

In this study, after the docking calculations were performed, ligands AAn0, AAn1, AAn2, and AAn3 were selected, as they exhibited a root-mean-square deviation (RMSD) of 0.00 Å. These ligands were then re-docked into the structure of *α*-glucosidase alongside acarbose, which also served as a positive control in the experimental assays. Figure S7 illustrates a partial superposition of the ligands (AAs and acarbose) within the same catalytic pocket. First, it indicated that both acarbose and the anacardic acids occupy the same catalytic pocket. Second, it suggested that these ligands (AAs) may share similar binding modes, which is essential for understanding their interaction mechanisms. Lastly, the proximity of the docked poses to the same binding site as the antidiabetic drug further supported the validity of the docking methodology employed.

In terms of binding affinity, acarbose was the most potent among the commonly used α-glucosidase inhibitors, with a binding affinity of −8.4 kcal/mol, which is higher than miglitol (−7.7 kcal/mol) and voglibose (−7.0 kcal/mol). This enhanced binding affinity makes acarbose more effective at inhibiting α-glucosidase. Three-dimensional visualizations (Figures S7–S10) revealed that acarbose, miglitol, voglibose, and anacardic acids (AAn1, AAn2, AAn3) all bound to the same pocket in α-glucosidase (PDB ID: 4J5T), forming hydrogen bonds with key residues, such as ASP568, ASP392, and TRP391. The fact that these compounds bound to the same pocket and formed hydrogen bonds with the same critical residues suggests that they share a similar binding mechanism. This indicates that they may probably compete for the same active site within the enzyme. Consequently, these inhibitors likely work through a comparable mode of action, targeting the catalytic site of α-glucosidase to inhibit its activity and reduce postprandial glucose levels.

This mechanism of action is particularly important for managing long-term glucose control, and acarbose is widely regarded as the first-line treatment for type 2 diabetes. However, while acarbose is effective at reducing postprandial glucose levels, especially in the 2 h postprandial period [71], it is often associated with a higher incidence of gastrointestinal side effects, such as bloating, gas, and diarrhea. Miglitol offers a valuable alternative with a lower risk of gastrointestinal discomfort, though it is slightly less potent than acarbose. Still, miglitol plays an important role in controlling blood glucose levels, particularly for patients who are more sensitive to gastrointestinal side effects. Voglibose, while less effective at controlling long-term blood glucose spikes, presents a better safety profile with fewer gastrointestinal issues than acarbose. It is a suitable option for patients seeking a gentler alternative with fewer side effects [72,73].

#### 3.3.1. Assessing AAn0-Binding Affinity: Effects of the Aromatic Head and Lack of Double Bonds in the Aliphatic Chain on In Silico Docking to the α-Glucosidase Catalytic Site Compared with Those of Acarbose

Figure 3 reveals that the binding modes of anacardic acids AAn1 and AAn0 in the active site were strikingly similar. This representation depicts the 3D and 2D binding patterns of AAn1-8*E* (a), AAn1-8*Z* (b), and AAn0 (c), showcasing a shared combination of noncovalent interactions involving the anacardic acids and specific amino acid components in the *α*-GLU enzyme’s catalytic pocket. These similar binding modes suggest that the enzyme exhibited comparable recognition of the anacardic acid ligands.

In an in silico simulation, AAn0 (Figure 3c), which lacks a double bond in its 15-carbon aliphatic chain, showed an interaction affinity of −8.6 kcal mol^−1^. In comparison, acarbose, a reference inhibitor, exhibited a slightly lower binding affinity of −8.4 kcal mol^−1^. This small difference of 0.2 kcal mol^−1^ suggests that AAn0 may bind slightly more strongly to the target protein than acarbose. These findings suggest that AAn0 may inhibit the enzyme in a manner comparable to acarbose, highlighting the important role of the 1,2,6-trisubstituted benzene moiety in this process. Earlier research demonstrated that AAn0 (6-pentadecylsalicylic acid) effectively inhibited *α*-glucosidase [74].

Figure 3c presents 2D and 3D representations of the noncovalent binding interactions between AAn0 and the amino acid residues in the catalytic pocket of the *α*-glucosidase enzyme. This figure illustrates the nonpolar interactions between the saturated aliphatic moiety of AAn0 and the residues PHE385 and PRO441.

Additionally, three of the most favorable hydrogen-bonding interactions involved the hydroxyl and carbonyl groups of the headgroup with GLY566 (AA-O-H•••O=C-GLY), TRP710 (indole ring-N-H•••O=C-AA), and TRP710 (indole ring-N-H•••O-AA). These hydrogen-bonding interactions are crucial for molecular recognition and enzymatic activity, as they contribute to the stabilization of the enzyme–substrate complex. The spatial separation between the atoms forming a hydrogen bond is critical, as it indicates the bond’s strength. Generally, a strong hydrogen bond has a distance of less than 3 Å [75], which correlates with a more stable and favorable interaction.

Collectively, the observed noncovalent interactions facilitated the formation of the enzyme–ligand complex, ultimately leading to enzymatic inhibition [76,77]. Therefore, in light of the results of the in silico studies, we conclude that the C=C double bond is not essential for the inhibitory activity of anacardic acids; rather, the –OH and –COOH groups in the trisubstituted benzene ring are critical for their anti-*α*-glucosidase activity.

#### 3.3.2. Investigating the Impact of Double Bonds and Stereochemistry on the In Silico Docking of Anacardic Acids to the Catalytic Site of α-Glucosidase

In this investigation, we performed five key comparisons to investigate the effects of double bonds and stereochemistry on the binding of anacardic acids to *α*-GLU: AAn0 (without a double bond) vs. AAn1-*E* (monoene), AAn1-*E* vs. AAn1-*Z* (stereoisomers of monoenes), AAn1-*E* (monoene) vs. AAn2-8*E*, 11*E* (dieno), AAn2-8*E*, 11*E* (dieno) vs. AAn2-8*Z*, 11*Z* (dieno), and AAn2-8*Z*, 11*E* (dieno) vs. AAn3-8*Z*, 11*E* (trieno). These comparisons aimed to elucidate how structural variations influence docking efficiency and overall binding affinity.

Initially, the comparison between AAn0 (Figure 3c, −8.6 kcal mol^−1^) and AAn1-*E* (Figure 3a, −9.9 kcal mol^−1^) indicated that the presence of a covalent C=C bond in the aliphatic chain may be associated with increased inhibitory activity. However, the binding affinity of AAn1-*Z* (Figure 3a, −8.9 kcal mol^−1^) was comparable to that of AAn0 (−8.6 kcal mol^−1^). This finding suggested that while the presence of a double bond may increase biological activity, this increase was also influenced by its stereochemistry.

A comprehensive examination of the interactions in the catalytic site of the 4J5T *α*-GLU enzyme revealed notable variations between the *E* and *Z* geometric isomers regarding hydrophobic interactions. Specifically, hydrophobic interactions were observed with the aromatic residue PHE385 in AAn1-*E*, whereas no such interactions were found in AAn1-*Z* (Figure 3a,b). Furthermore, as mentioned previously, three of the most favorable hydrogen-bonding interactions were identified with GLY566, TRP391, and TRP710. It appears that the observed hydrophobic interaction assisted in orienting the aromatic headgroup closer to these residues. Research on the *E* and *Z* isomers of monoene anacardic acids (AAn1-*E*at −9.9 and AAn1-*Z*at −8.9 kcal mol^−1^) revealed a 1 kcal mol^−1^ difference in binding affinity, indicating that the *E* stereochemistry was more favorable for binding. Investigating how these different *E*/*Z* isomers interact with the molecular target is essential for understanding their biological efficacy [22,78,79].

To enhance the discussion, it is important to compare the binding affinities of the monoene compound AAn1-*E* (−9.9 kcal mol^−1^, Figure 3a) and the diene compound AAn2-8*E*,11*E* (−10.2 kcal mol^−1^, Figure 4a). Interestingly, despite the diene showing a more favorable binding energy, the experimental results indicated that the monoene AAn1 had a stronger inhibitory effect in these assays, with an IC_50_ of 1.78 ± 0.08 μg mL^−1^, compared with 1.99 ± 0.76 μg mL^−1^ for the diene.

Specific hydrophobic interactions with the aromatic residue PHE385 were observed in monoene AAn1-*E* (Figure 3a), whereas such interactions were absent in dienes and trienes (Figure 4 and Figure 5). This suggests that monoene AAn1-*E* might engage in more favorable interactions with PHE385, making these interactions crucial for effective binding and enzyme inhibition. Thus, the combination of binding energy, ligand conformation, and specific molecular interactions has emerged as a critical factor influencing the inhibitory efficacy of these compounds [65,67].

This study demonstrated that anacardic acid inhibitors can effectively bind to the substrate-binding pocket of 4J5T *α*-glucosidase, positioning a warhead near GLY566, TRP710, and TRP391, which we propose are crucial catalytic residues. We hypothesize that the hydrophobic interaction with PHE385 contributes to enhanced biological activity, influenced by the *E*/*Z* geometry of the carbon-(sp^2^)–carbon-(sp^2^) double bond. Importantly, our findings represent a novel contribution to the field and open up opportunities for further research on how the chemical structure influences α-glucosidase inhibitory activity.

### 3.4. In Silico ADMET and Drug-Likeness Evaluation

Given the importance of understanding the pharmacokinetic profile and toxicity in the early phases of drug discovery, a study was conducted to predict the ADMET and drug-likeness properties of anacardic acids (AAn1, AAn2, and AAn3) via the online tools pkCSM [80], SwissADME [81], and Protox II [49,82].

Drug-likeness allows for a qualitative evaluation of whether a molecule has the potential to be developed into an oral drug in terms of bioavailability. The method was developed based on structural and physicochemical inspections of oral drug candidates. Thus, drug-likeness analysis was performed using rules that establish thresholds for physicochemical properties, with Lipinski’s rule (Rule of 5) being the pioneer and most popular [81,83,84]. In addition to the Rule of 5, the AAs under study were evaluated via the Veber, Ghose, Muegge, and Egan methods [85]. The results of this analysis as well as the physicochemical properties and water solubility of the AAs are presented in Table 3.

The compounds showed only one violation for each method. In all the filters, violations are related to lipophilicity, with the exception of the Veber method, in which the violation is due to the number of rotary bonds above the desirable level (≤10). The Log *P*_o/w_ values predicted for AAs by the different predictive models showed that lipophilicity decreased as the number of double bonds increased, with triene anacardic acid (AAn3) being the least lipophilic. The molecule became less linear as the number of double bonds increased, resulting in an increase in polarity [25]. Regarding aqueous solubility, the Log S values predicted by the Ali Log S method of the SwissADME tool indicated that AAs were poorly soluble in water, which will limit their absorption and consequently their bioavailability. Some strategies are already being explored to overcome this limitation and improve the bioavailability [31,86].

The results of the prediction of pharmacokinetic properties performed by the pkCSM web server can be seen in Table 4. Regarding parameter absorption, the following descriptors were used: Caco-2 permeability, P-glycoprotein (P-gp) substrate and inhibitor, human intestinal solubility, and skin permeability. Oral drug absorption is largely estimated using human colon adenocarcinoma cells (Caco-2) [87]. From the Caco-2 permeability test, it is possible to obtain P_app_, the apparent permeability coefficient, which is a parameter used as an indicator of a compound’s bioavailability [84]. A predicted Log P_app_ value greater than 0.90 at 10^−6^ cm/s indicates high permeability [80], which was the case for the AAs studied (Table 4). The results were also positive for the intestinal absorption parameter, with the lowest percentage absorbed (88%) presented by AAn3.

P-glycoprotein (P-gp) is a glycosylated transmembrane protein present throughout the body that is capable of expelling (active transport) a wide range of substances out of cells. In the intestine, for example, P-gp can affect drug absorption by ejecting it back into the gastrointestinal lumen. According to the prediction, the AAs in the study are neither inhibitors nor substrates of P-gp. Therefore, the absorption of anacardic acids will not be affected by the action of this transporter, and there will be no risk of drug–drug interactions if AAs are co-administered with P-gp substrates [88].

Skin permeability, which is an important parameter to consider with respect to transdermal drug delivery, is considered relatively low if the compound presents a log Kp > −2.5 [80]. All the compounds evaluated presented log Kp values outside this range.

The distribution process was evaluated by the predicted results for the fraction unbound (human), volume of distribution at steady state (VDss), and blood–brain barrier (BBB) and central nervous system (CNS) permeability. In plasma, most drugs will have a fraction bound to serum proteins and an unbound fraction (Fu) [80]. According to the free drug theory, unbound drugs should indicate the concentration of the drug in the tissues that can bind to the target receptor [89,90]. As shown in Table 4, the acids presented low predicted values for Fu.

The volume of distribution parameter provides information about the distribution of a drug in tissues [91]. The low VDs of AAs (Table 4) indicated that their distribution was greater in the bloodstream [92]. In terms of the BBB, compounds that can easily cross it have log BB > 0.3, and those that have a poor distribution to the brain have a logBB < −1. CNS permeability, in turn, is assessed on the basis of logPS values. Thus, compounds that can penetrate the CNS have logPS > −2, and those that cannot penetrate the CNS have logPS < −3 [80]. As the logBB and logPS values of the AAs (Table 4) were outside these ranges, it was not possible to conclude from the prediction whether the AAs could reach the CNS. However, in vivo analyses revealed the pharmacological activities of AAs in the CNS, such as anxiolytic activity mediated by the GABA receptor [18] and neuroprotective activity in a rotenone model of Parkinson’s disease [93,94].

Metabolism was assessed to determine whether anacardic acids are inhibitors of cytochrome P450 (CYP) 1A2, CYP2C19, and CYP2C9 isoforms and inhibitors and/or substrates of the CYP2D6 and CYP3A4 isoforms. Many drugs are metabolized by CYP450 enzymes [80]. When metabolism inhibition occurs because drugs compete for metabolism by the same CYP enzyme, an unexpected increase in the plasma concentration of one or both drugs may occur, which can lead to severe adverse effects [95]. According to the predicted results for the metabolic profile (Table 4), all AAs were non-inhibitors of the CYP isoenzymes considered, and AAn3 was a potential substrate of CYP3A4.

For the excretion parameter, two descriptors were considered: total clearance and renal OCT2 substrate. Regarding the organic cation transporter 2 (OCT2), the three acids were classified as non-substrates. Therefore, it is expected that this renal transporter does not participate in the excretion process of anacardic acids and that adverse effects will not occur if OCT2 inhibitors are co-administered [80]. In terms of total clearance, a high value means that the excretion process of a given medication will be rapid [96]. The predicted total clearance log (CL_tot_) values indicated that triene (1.717) had a faster excretion process than diene (1.533) and monoene (1.573).

The in silico toxicity of AAs was evaluated in terms of acute toxicity (LD_50_), mutagenicity (Ames), cardiotoxicity (hERG), hepatotoxicity, skin sensitization, and maximum tolerated dose (human). According to the predicted results (Table 4), the AAs studied were not hERG I/II inhibitors and, therefore, were not cardiotoxic. They were also not mutagenic or hepatotoxic and did not have the potential for skin sensitization. The maximum recommended tolerated dose of AAs can be considered low (MRTD ≤ 0.477) [80]. The median lethal dose (LD_50_) is a way of expressing acute toxicity and represents the predicted dose that results in the death of half of a test group [97]. The LD_50_ value of AAs calculated by Protox II was 1000 mg kg^−1^ weight, with a 68.5% average accuracy prediction. Therefore, the compounds belonged to toxicity class IV (300 < LD_50_ ≤ 2000) of the GHS toxicity classification (Table 5). Compounds considered to be of low toxicity have LD_50_ > 2000 mg kg^−1^ [97]. Thus, the results suggested that strategies to increase the LD_50_ of AAs will be necessary to increase their potential for clinical applications.

## 4. Conclusions

This study underscored the significant potential of anacardic acids (AAs) derived from cashew nut shell liquid (CNSL) as novel inhibitors of *α*-glucosidase, indicating an opportunity for diabetes therapy research. We successfully identified and characterized three distinct AAs—monoene, diene, and triene—as well as their combined mixture, demonstrating that all exhibited a strong ability to inhibit α-glucosidase, presenting IC_50_ values markedly lower than the standard drug acarbose. Among them, the monoene (AAn1: 2-hydroxy-6-(pentadec-8-en-1-yl)benzoic acid) proved to be the most potent inhibitor. Molecular docking analysis revealed that the polar groups in the 1,2,6-trisubstituted benzene ring were crucial for forming stable enzyme–ligand complexes, while the double bond found in the C-6 alk(en)yl side chain enhanced the inhibitory effect, though it was not essential. This is the first study to report these findings, laying the groundwork for future research aimed at understanding the structure–activity relationship of these compounds. Pharmacokinetic analyses suggested that these AAs possess moderately favorable drug-like properties, underscoring their potential for therapeutic use. In conclusion, the efficacy of anacardic acids as *α*-glucosidase inhibitors not only reinforces the importance of renewable natural resources in drug development but also warrants further investigation—particularly through in vivo studies—to validate their therapeutic potential and optimize their use in diabetes management.

## Figures and Tables

**Table 1:** MS data, purity (%), ^1^H and ^13^C chemical shifts (*δ*), coupling constants (*J* (Hz)), multiplicity, and long-range heteronuclear ^1^H-^13^C HMBC correlations for the anacardic acids (26 °C, DMSO-*d*_6_).

Numbered Structure/Chemical Formula	*δ*^1^H (ppm), (Multiplicity, *J* (Hz))	*δ*^13^C (ppm) HSQC (H→C)	C-n HMBC (H→C)	UPLC/MS Ions [M-H]^−^ and [M-CO_2_]^−^ ^e^	Purity (%) ^f^	Reference
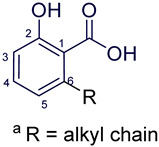	*1,2,6-trisubstituted benzene ^a,b^*					[55]
	*δ 11.09 (s)*	171.2 (COOH) ^c^				
		110.1 (C-1)				
		165.9 (C-2)				
	*δ* 6.54 (d, 7.7, 1H-3) ^c^	115.2 (C-3)	C-2			
	*δ* 7.02 (t, 7.7, 1H-4) ^c^	136.3 (C-4)	C-2			
	*δ* 6.49 (d, 7.7, 1H-5) ^c^	126.4 (C-5)	C-2, C-4			
		142.9 (C-6)				
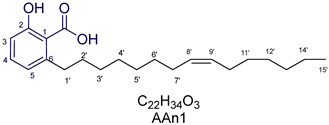	*Alkyl chain—AAn1*			*m*/*z* 345.2397	92.6	[56]
	*δ* 2.59 (t, 7.2 Hz, 2H-1′) ^c^	34.3 (C-1′)	C-5′, C-6′	*m*/*z* 301.2517		
	*δ* 1.46 (t, 7.2 Hz, 2H-2′) ^c^	31.5 (C-2′)	C-6′			
	*δ* 1.25 (m, 2H-3′)	31.4(C-3′)				
	*δ* 1.21 (m, 2H-4′)	31.4(C-4′)				
	*δ* 1.22 (m, 2H-5′)	31.6(C-5′)				
	*δ* 1.30 (m, 2H-6′)	28.9 (C-6′)				
	*δ* 1.97 (qt, 7.1 Hz, 4H-7′,10′)	28.5 (C-7′)	C-8′			
	*δ* 5.34 (m, 1H-8′) ^d^	130.1 (C-8′,9′)				
	*δ* 5.34 (m, 1H-8′) ^d^	130.1 (C-8′,9′)				
	*δ* 1.23 (m, 2H-11′)	29.9 (C-11′)	C-9′			
	*δ* 1.26 (m, 2H-12′)	29.8 (C-12′)				
	*δ* 1.24 (m, 2H-13′)	31.9 (C-13′)				
	*δ* 1.26 (q, 7.3 Hz,2H-14′)	22.2 (C-14′)				
	*δ* 0.85 (t, 7.3 Hz,3H-15′)	14.0 (C-15′)	C-14′, C-13′,C-12′			
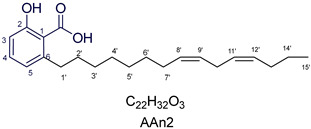	*Alkyl chain—AAn2*			*m*/*z* 343.2243	95.5	[57]
	*δ* 2.61 (t, 7.0 Hz, 2H-1′) ^c^	35.0 (C-1′)	C-5, C-6	*m*/*z* 299.2364		
	*δ* 1.47 (t, 7.0 Hz, 2H-2′) ^c^	31.5 (C-2′)				
	*δ* 1.24 (m, 2H-3′)	31.9 (C-3′)				
	*δ* 1.23 (m, 2H-4′)	31.7 (C-4′)	C-2			
	*δ* 1.23 (m, 2H-5′)	31.0 (C-5′)				
	*δ* 1.31 (qt, 7.0 Hz, 2H-6′)	29.0 (C-6′)				
	*δ* 2.01(qt, 7.0 Hz, 4H-7′,10′)	28.9 (C-7′,10′)				
	*δ* 5.37 (m, 1H-8′) ^d^	130.0 (C-8′)	C-8’			
	*δ* 5.34 (m, 1H-9′)	129.3 (C-9′)				
	*δ* 2.81 (qt, 7.0 Hz, 2H-10′)	25.8 (C-10′)	C-9′			
	*δ* 5.34 (m, 1H-11′) ^d^	129.0 (C-11′)				
	*δ* 5.37(m, 1H-12′) ^d^	129.8 (C-12′)				
	*δ* 1.99 (m, 2H-13′)	32.1 (C-13′)	C-15′, C-12′			
	*δ* 1.38 (q, 7.3 Hz,2H-14′)	23.3 (C-14′)				
	*δ* 0.85 (t, 7.3 Hz,3H-15′)	14.7 (C-15′)				
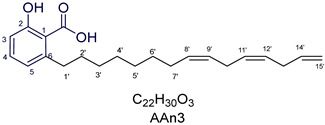	*Alkylchain—AAn3*			*m*/*z* 341.2093	99.1	[57]
	*δ* 2.59 (t, 6.9 Hz 2H-1′) ^c^	34.9 (C-1′)		*m*/*z* 297.2213		
	*δ* 1.49 (t, 6.9 Hz, 2H-2′) ^c^	25.0 (C-2′)				
	*δ* 1.46 (m, 2H-3′)	31.2 (C-3′)				
	*δ* 1.31 (m, 2H-4′)	30.1 (C-4′)				
	*δ* 1.29 (m, 2H-5′)	29.9 (C-5′)				
	*δ* 1.25 (m, 2H-6′)	28.6 (C-6′)				
	*δ* 1.99 (m, 2H-7′)	28.1 (C-7′)				
	*δ* 5.28 (d, 6.9 Hz, 1H-8′) ^d^	130.0 (C-8′)				
	*δ* 5.33 (d, 6.9 Hz, 1H-9′) ^d^	127.8 (C-9′)				
	*δ* 2.79 (t, 6.9 Hz, 2H-10′)	25.4 (C-10′)	C-11’			
	*δ* 5.33 (m, 1H-11′) ^d^	128.0 (C-11′)				
	*δ* 5.33 (m, 1H-12′) ^d^	127.9 (C-12′)				
	*δ* 2.83 (qt, 7.4 Hz, 2H-13′)	25.8 (C-13′)	C-12′			
	*δ* 5.75 (d, 1H-14′)	137.8 (C-14′)				
	*δ* 4.99 (dd, 17.0 and 10.0 Hz, 1H-15′) ^d^	115.2 (C-15′)	C-14′			
	*δ* 5.01 (dd, 1.9 and 10.0 Hz, 1H-15′) ^d^					

^a^ All AAs have similar aromatic chemical shifts, which are unaffected by variations in unsaturation. ^b^ Abbreviations for the resonance multiplicity of protons include singlet (s), doublet (d), doublet of doublets (dd), triplet (t), quartet (q), quintet (qt), and multiplet (m). C-n refers to the “n” numbering, as observed at each carbon in the drawn anacardic acid structure. ^c^ Assignments were performed via 1D and 2D NMR techniques, specifically via ^1^H-1H COSY, ^1^H-^13^C HMBC, and ^1^H-^13^C HSQC cross-peaks (chemical shift correlations). ^d^ Relative magnitudes of ^1^H-^1^H coupling constants: *J*_cis_ = 7–10 Hz, *J*_trans_ = 12–18 Hz, and *J*_geminal_ = 1–3 Hz. ^e^ The *m*/*z* error measurements obtained with the QToF instrument show values of 9.26 ppm for AAn1, −8.74 ppm for AAn2, and −6.75 ppm for AAn3. The *m*/*z* error (ppm) represents the difference between the theoretical and measured *m*/*z* values. ^f^ Purity via HPLC/UV-Vis.

**Table 2:** Predicted values of affinity (kcal mol^−1^) for anacardic acid isomers present in the cashew nut shell (*A. occidentale*) and the reference drug docked at the active site of *α*-glucosidase.

*n*	*N*	Anacardic Acid Structures Acronym: AAn(1, 2 or 3)-*N*(*E* or *Z*) ^a^	Binding Affinity (kcal mol^−1^)	Amino Acid Residue (Enzyme–Ligand Binding Distance in Å)
	
		*(15:1)-anacardic acid isomers (AAn1)*		
**1**	8	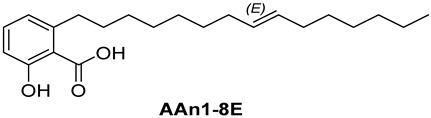	**−9.9**	**GLY566 (2.1 Å) ^c^**, TRP391 (3.1 Å), ASP392 (3.9 Å), TRP710 (3.9 Å), **PHE385 (3.0 Å),**
**1**	8	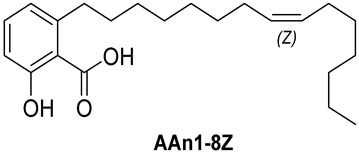	−8.9	**GLY566 (3.7 Å),** TRP391 (3.0 Å), ARG392 (3.0 Å) ^c^, TRP710 (3.0 Å), ASP568 (3.0 Å)
		*(15:2)-anacardic acid isomers (AAn2)*		
**2**	8, 11	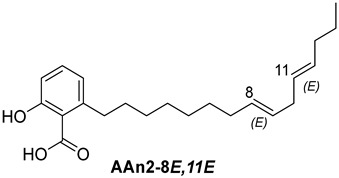	**−10.2**	**GLY566** (1.9 Å) ^c^, ASP568 (1.9 Å), TRP710 (3.0 Å), PHE444 (2.8 Å), LEU563 (3.1 Å)
**2**	8, 11	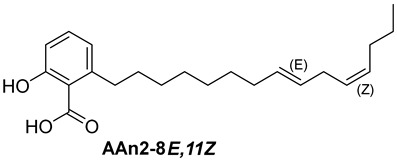	−9.4	**GLY566** (2.5 Å) ^c^, ASP568 (3.0 Å), TRP710 (3.9 Å)
**2**	8, 11	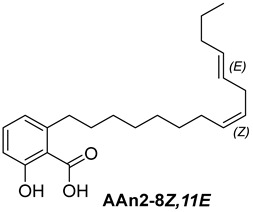	−9.5	**GLY566** (3.0 Å) ^c^, ASP568 (2.7 Å), TRP710 (2.9 Å), PHE444 (2.8 Å)
**2**	8, 11	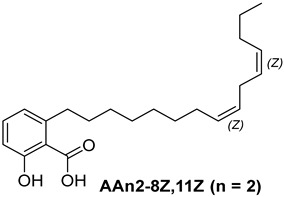	−8.9	**GLY566** (3.0 Å) ^c^, ASP568 (2.7 Å), TRP710 (2.9 Å)
		*(15:3)-anacardic acid isomers (AAn3)*		
**3**	8, 11	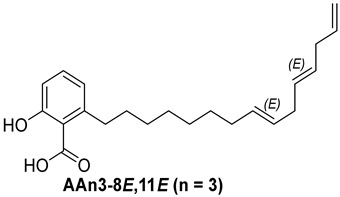	−9.2	**GLY566** (2.4 Å) ^c^, TRP391 (3.1 Å), ARG392 (2.9 Å), TRP710 (3.0 Å), PHE444 (3.1 Å), LEU563 (4.0 Å),
**3**	8, 11	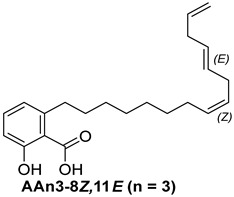	−9.2	**GLY566** (1.9 Å) ^c^, ASP568 (3.0 Å), TRP710 (3.0 Å)
**3**	8, 11	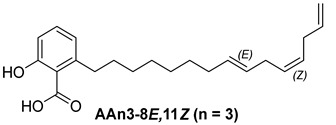	−9.0	**GLY566** (2.2 Å), ASP568 (3.2 Å), TRP710 (2.8 Å)
**3**	8, 11	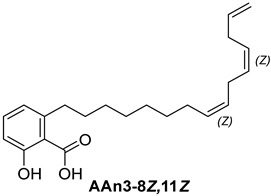	−8.4	**GLY566** (2.7 Å), ASP568 (3.1 Å), TRP710 (3.0 Å), PHE444 (3.0 Å)
**0**	0	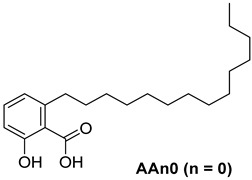	−8.6 ^b^	**GLY566** (4.7 Å) ^c^, ASP568 (3.7 Å), TRP710 (4.0 Å), ARG428 (4.3 Å), ARG428 (3.6 Å), PHE385 (2.2 Å), PRO441 (2.8 Å),
		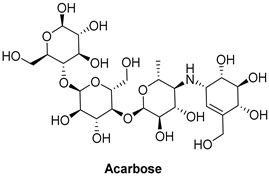	−8.4	**GLY566** (1.9 Å) ^c^, ASP568 (2.0 Å), ARG387(2.3 Å), **PHE384** (1.5 Å), LYS439 (1.9 Å), GLY383 (2.1 Å), TRP710 (2.4 Å), TRP391(2.4 Å)
		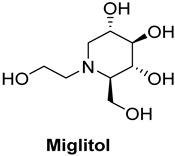	−7.7	ASP392 (1.9 Å) ASP568 (2.8 Å) TRP391 (2.0 Å)
		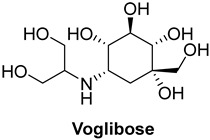	−7.0	ASP392 (1.7 Å) ASP568 (3.0 Å) TRP391 (2.3 Å)

^a^ AAn (0, 1, 2, or 3)-(N–E or Z), where “AA” refers to anacardic acid, “n” indicates the number of double bonds, and “N–E or Z” provides information about the position of the alkyl chain (N = C8 and/or C11) and configuration of the double bonds within the compound (*E* or *Z*). ^b^ The compound 15:0-anacardic acid (AAn0), lacking a double bond in its alkyl chain, was evaluated solely through in silico methods. ^c^ AAn0 had a hydrogen bond distance of 4.7 Å to GLY566, which was considered unfavorable and included only for comparison. Noncovalent interaction distances of up to 3.0 Å were deemed relevant.

**Table 3:** Physicochemical properties and drug-likeness results predicted for anacardic acids by SwissADME.

	Anacardic Acids		
	AAn1	AAn2	AAn3
Physicochemical properties			
*Mol weight* (g mol^−1^)	346.50	344.49	342.47
*Number of H-bond acceptors*	3	3	3
*Number of H-bond donors*	2	2	2
*Number of rotatable bonds*	14	13	13
*Molecular refractivity*	107.21	106.74	106.27
*Topological polar surface area* (Å^2^)	57.53	57.53	57.53
*Log Po/w* (*MLOGP*)	4.85	4.77	4.69
*Log Po/w* (WLOGP)	6.50	6.28	6.05
*Log Po/w* (XLOGP3)	8.55	7.86	7.6
Drug-likeness filter			
*Lipinski*	1 violation: MLOGP > 4.15	1 violation: MLOGP > 4.15	1 violation: MLOGP > 4.15
*Ghose*	1 violation: WLOGP > 5.6	1 violation: WLOGP > 5.6	1 violation: WLOGP > 5.6
*Veber*	1 violation: Rotors > 10	1 violation: Rotors > 10	1 violation:Rotors > 10
*Egan*	1 violation: WLOGP > 5.88	1 violation: WLOGP > 5.88	1 violation: WLOGP > 5.88
*Muegge*	1 violation: XLOGP3 > 5	1 violation: XLOGP3 > 5	1 violation: XLOGP3 > 5
Water solubility (*Ali Log S*)	**−9.63**	**−8.92**	**−8.65**

**Table 4:** ADMET prediction data of anacardic acids by pkCSM.

Model Name	Unit	Predicted Value for Anacardic Acids (AA) ^a^		
		*(15:1)-AA*	*(15:2)-AA*	*(15:3)-AA*
**Absorption**				
Caco-2 permeability	Numeric (log P_app_ in 10^−6^ cm s^−1^)	1.133	1.145	1.269
Intestinal absorption (human)	Numeric (% Absorbed)	94.344	94.85	88.009
Skin permeability	Numeric (log Kp)	−2.735	−2.735	−2.662
P-glycoprotein substrate	Categorical (Yes/No)	No	No	No
P-glycoprotein I inhibitor	Categorical (Yes/No)	No	No	No
P-glycoprotein II inhibitor	Categorical (Yes/No)	No	No	No
**Distribution**				
VDss (human)	Numeric (log L kg^−1^)	−1.309	−1.345	−0.235
Fraction unbound (human)	Numeric (Fu)	0.097	0.096	0.009
BBB permeability	Numeric (log BB)	−0.051	−0.024	−0.416
CNS permeability	Numeric (log PS)	−2.458	−2.405	−2.374
**Metabolism**				
CYP2D6 substrate	Categorical (Yes/No)	No	No	No
CYP3A4 substrate	Categorical (Yes/No)	No	No	Yes
CYP1A2 inhibitior	Categorical (Yes/No)	No	No	No
CYP2C19 inhibitior	Categorical (Yes/No)	No	No	No
CYP2C9 inhibitior	Categorical (Yes/No)	No	No	No
CYP2D6 inhibitior	Categorical (Yes/No)	No	No	No
CYP3A4 inhibitior	Categorical (Yes/No)	No	No	No
**Excretion**				
Total clearance	Numeric (log mL/min/kg)	1.533	1.573	1.717
Renal OCT2 substrate	Categorical (Yes/No)	No	No	No
**Toxicity**				
AMES toxicity	Categorical (Yes/No)	No	No	No
Max. tolerated dose (human)	Numeric (log mg/kg/day)	0.134	0.143	0.222
hERG I inhibitor	Categorical (Yes/No)	No	No	No
hERG II inhibitor	Categorical (Yes/No)	No	No	No
Hepatotoxicity	Categorical (Yes/No)	No	No	No
Skin sensitization	Categorical (Yes/No)	No	No	No

Apparent permeability coefficient (P_app_); HERG: human ether-related gene channel; MDCK: Mandin Darby Canine Kidney. ^a^ Nomenclature: (15:1)-AA, (15:2)-AA, and (15:3)-AA for AAn1 (monoene), AAn2 (diene), and AAn3 (triene), respectively.

**Table 5:** Acute oral toxicity prediction via the Protox II web server.

Anacardic Acid	Oral Toxicity Prediction Results			
	Predicted LD_50_ (mg/kg)	Predicted Toxicity Class ^a^	Average Similarity (%)	Prediction Accuracy (%)
(15:1)-AA	1000	4	70.11	69.26
(15:2)-AA	1000	4	67.6	68.07
(15:3)-AA	1000	4	67.6	68.07

^a^ GHS toxicity classification—Class I: fatal if swallowed (LD_50_ ≤ 5), Class II: fatal if swallowed (5 < LD_50_ ≤ 50), Class III: toxic if swallowed (50 < LD_50_ ≤ 300), Class IV: harmful if swallowed (300 < LD_50_ ≤ 2000), Class V: may be harmful if swallowed (2000 < LD_50_ ≤ 5000), and Class VI: non-toxic (LD_50_ > 5000).

## Data Availability

The original contributions presented in the study are included in the article/Supplementary Materials, further inquiries can be directed to the corresponding author.

## Supplementary Materials

The following supporting information can be downloaded at: https://www.mdpi.com/article/10.3390/foods13244107/s1. Figure S1: Chromatographic profile of the sample mixture (mix) analyzed by UPLC–QTOF-MS^E^ in negative mode. Figure S2: Negative-ion ESI-MS spectra of (15:1)-anacardic acid. Figure S3: Negative-ion ESI-MS spectra of (15:2)-anacardic acid. Figure S4: Negative-ion ESI-MS spectra of (15:3)-anacardic acid. Figure S5: Two-dimensional representation of the binding interactions between acarbose and the amino acid residues at the active site of *α*-glucosidase (PDB ID: 4J5T). Figure S6: Three-dimensional representation of the docked pose of acarbose at the active site of *α*-glucosidase (PDB ID: 4J5T). Figure S7: 3D visualization of the re-docking results demonstrating the superposition of the binding modes of AAn0, AAn1, AAn2, and AAn3, together with the reference inhibitor acarbose. Figure S8: (a) 3D visualization showing the partial superimposition of the antidiabetic drugs voglibose (dark pink), miglitol (yellow), and the reference inhibitor acarbose (yellow). (b) Enzyme–ligand complex showing all drugs binding to the same α-glucosidase pocket. (c) 3D view showing miglitol, voglibose, and anacardic acids binding to the same pocket, forming hydrogen bonds with ASP568, ASP392, and TRP391. Figure S9: Three-dimensional representation of the binding interactions between miglitol (with carbon in blue in the center) and the amino acid residues at the active site of α-glucosidase (PDB ID: 4J5T). Figure S10: Three-dimensional representation of the binding interactions between voglibose (with carbon in yellow in the center) and the amino acid residues at the active site of *α*-glucosidase (PDB ID: 4J5T). Figures S11–S13: The ^1^H NMR spectra (600 MHz, DMSO-*d*_6_) of AAn1, AAn2, and AAn3. Figure S14: The ^1^H NMR spectrum (150 MHz, DMSO-*d*_6_) of AAn2. Figures S15–S17: ^1^H-^13^C HSQC contour maps (600/150 MHz, DMSO-*d*_6_) of AAn1, AAn2, and AAn3. Figure S18: ^1^H-^13^C HMBC contour map (600/150 MHz, DMSO-*d*_6_) of AAn2. Figure S19: ^1^H-^13^C COSY contour map (150/150 MHz, DMSO-*d*_6_) of AAn2. Table S1: Tukey’s multiple comparison test for differences in mean percentage inhibition data for the 95% confidence level.
