# Supplementary material for: Structural Characterization and In Vitro and In Silico Studies on the Anti-α-Glucosidase Activity of Anacardic Acids from Anacardium occidentale
Source: Foods. 2024 Dec 19;13(24):4107. doi: 10.3390/foods13244107 (PMC11675133; doi:10.3390/foods13244107)
Supplement: Supplementary file 1 [file foods-13-04107-s001.zip › foods-3302160-supplementary.pdf]

SUPPLEMENTARY MATERIAL

Figures and Tables

Structural characterization and in vitro and in silico studies on the anti-α-glucosidase activity of Anacardic acids from *Anacardium occidentale*

Ana Priscila Monteiro da Silva [1,2#], Gisele Silvestre da Silva [1#], Francisco Oiram Filho [1,2], Maria Francilene Souza Silva [3], Guilherme Julião Zocolo[1,4], Edy Sousa de Brito [5,*]

[1] Embrapa Agroindústria Tropical, 60511-110, Fortaleza, Ceará, Brazil
[2] Department of Chemical Engineering, UFC, Federal University of Ceará, Campus do Pici, Bloco 709, 60455-760, Fortaleza, CE, Brazil
[3] Research and Development of Medicines, Federal University of Ceará, Rua Coronel Nunes de Melo 1000, Rodolfo Teófilo, 60420-275, Fortaleza, Ceará, Brazil
[4] Embrapa Soja, 86085-981, Londrina, PR, Brazil
[5] Embrapa Alimentos e Territórios, 57020-050, Maceió, AL, Brazil
* Correspondence: edy.brito@embrapa.br
[#] These authors share equal contribution to this study.

Relevant results from Tukey's multiple comparison test

Table S1: Tukey multiple comparison test for differences of mean percentage inhibition data for 95 % of confidence level.

Tukey's multiple comparisons test	Mean Diff,	95,00% CI of diff,	Significant?	Summary	Adjusted P Value
AAn1 vs. AAn2	1,623	-6,764 to 10,01	No	ns	0,9551
**AAn1 vs. AAn3**	**13,61**	**5,219 to 21,99**	**Yes**	***	**0,0004**
**AAn1 vs. mix**	**15,44**	**7,053 to 23,83**	**Yes**	****	**<0,0001**
**AAn2 vs. AAn3**	**11,98**	**3,596 to 20,37**	**Yes**	**	**0,0022**
**AAn2 vs. mix**	**13,82**	**5,430 to 22,20**	**Yes**	***	**0,0004**
AAn3 vs. mix	1,834	-6,553 to 10,22	No	ns	0,937

Statistical significance: $^{ns}p > 0.05$, $^{*}p < 0.05$, $^{**}p < 0.01$, $^{***}p < 0.001$, $^{****}p < 0.0001$.

Chromatographic profile of mix (AAn1 +AAn2 +AAn3)

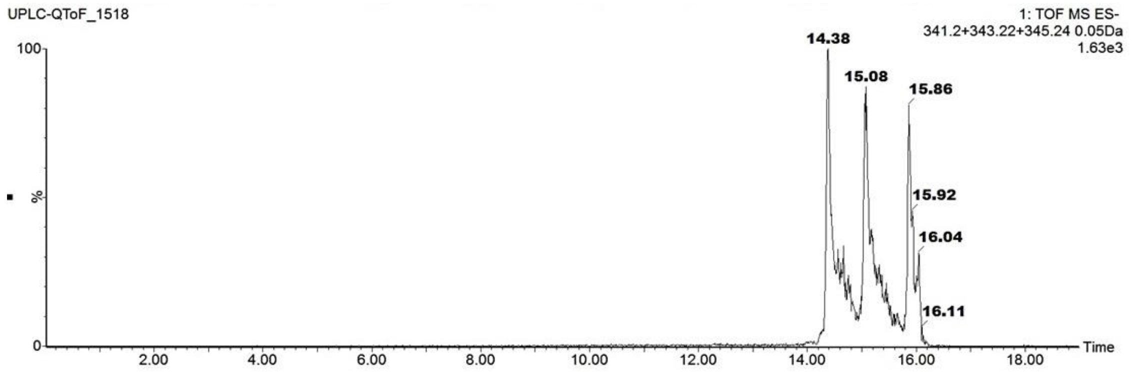

Figure S1: Chromatographic profile of sample mixture (mix) analyzed by UPLC–QTOF-MS^E in negative mode.

Negative ion ESI-MS spectra for AAn1, AAn2, and AAn3

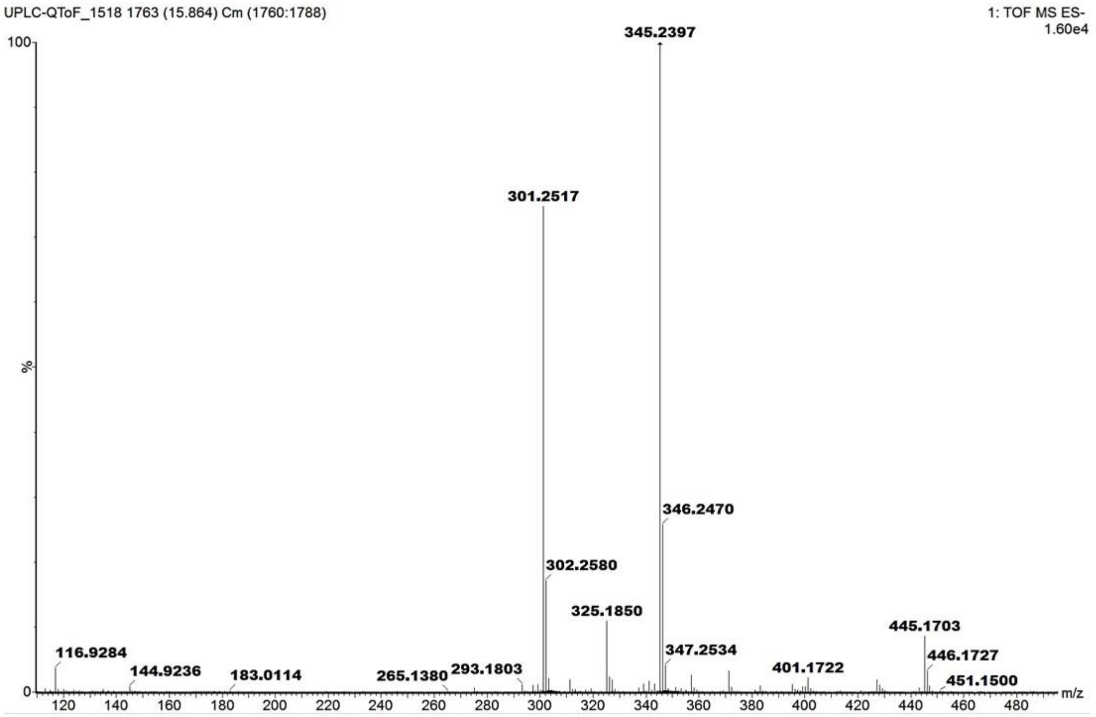

Figure S2: Negative ion ESI-MS spectra of (15:1)-Anacardic acid (AAn1, Exact mass: 345.2429 for $C_{22}H_{33}O_3$ $[M-H]^-$, calculated mass: 345.2397, mass error: 9.26 ppm).

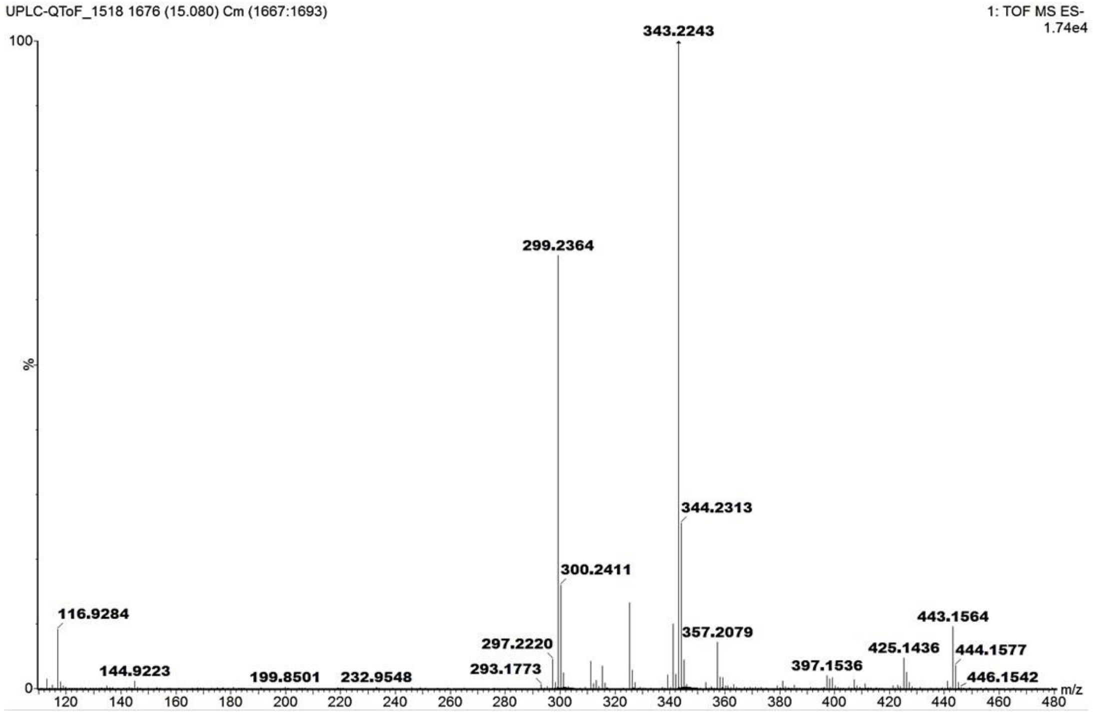

Figure S3: Negative ion ESI-MS spectra of (15:2)-Anacardic acid (AAn2, Exact mass: 343.2273 for $C_{22}H_{31}O_3$ $[M-H]^-$, found mass: 343.2243, mass error: -8.74 ppm).

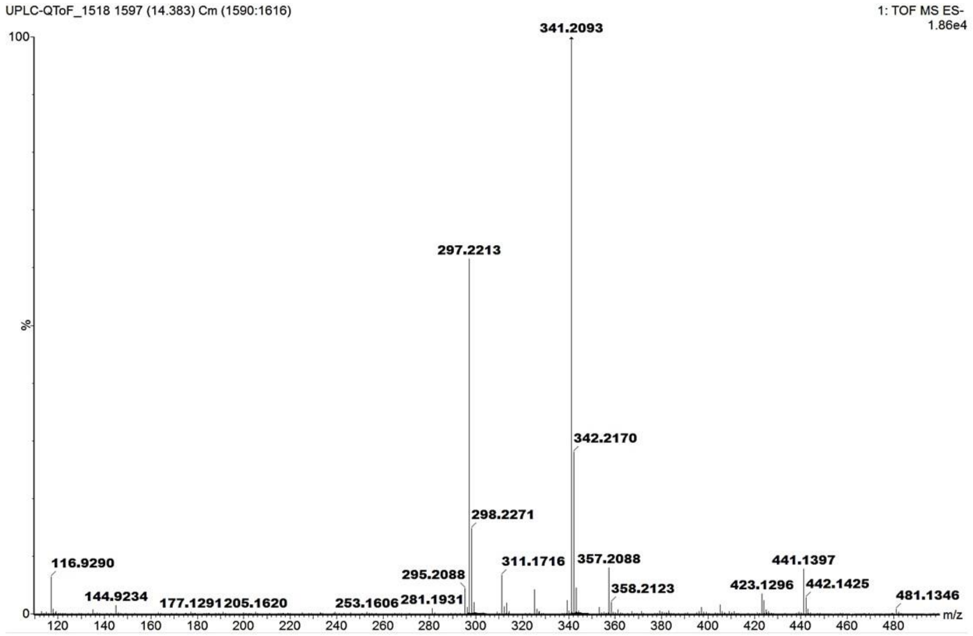

Figure S4: Negative ion ESI-MS spectra of (15:3)-Anacardic acid (AAn3, Exact mass: 341.2116 for $C_{22}H_{29}O_3$ $[M-H]^-$, found mass: 341.2093, mass error: -6.74 ppm).

Relevant molecular docking results for anacardic acids and other commercial drugs associated with diabetes, including acarbose, miglitol, and voglibose

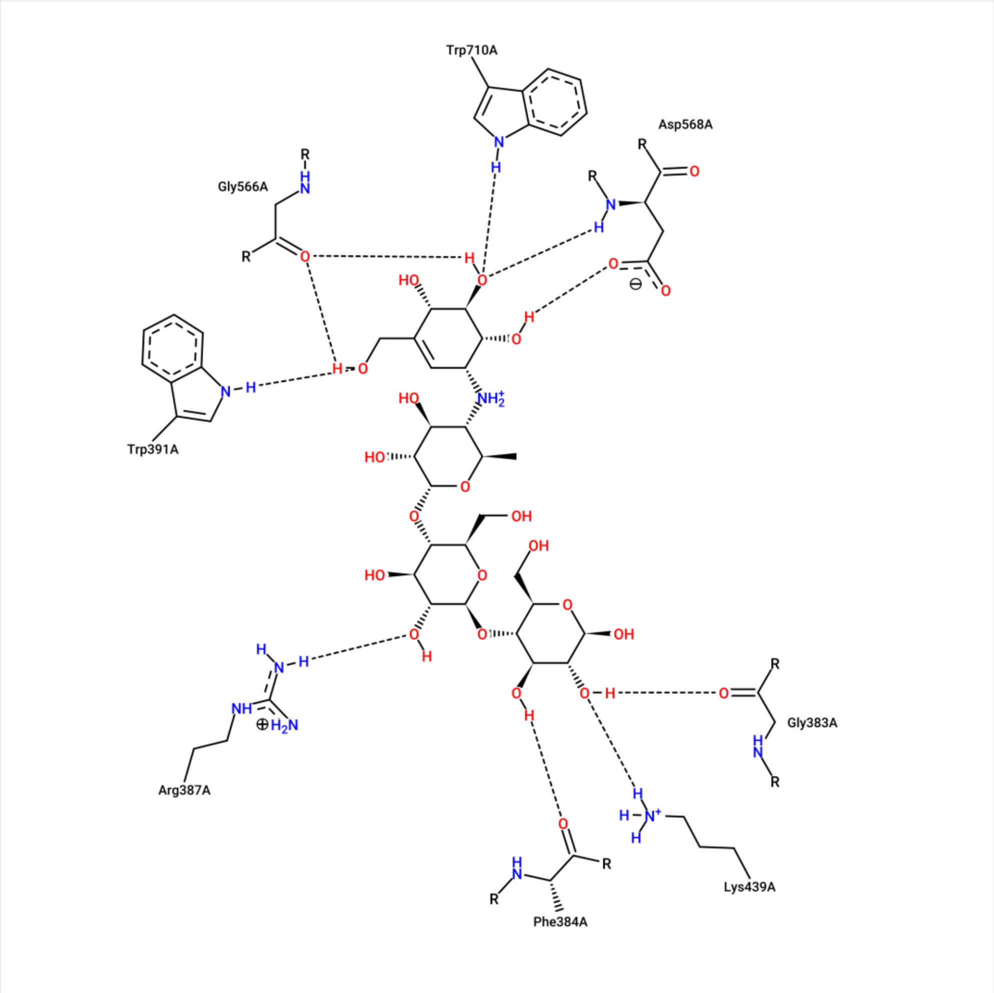

Figure S5: Two-dimensional representation of the binding interactions between acarbose and the amino acid residues at the active site of α-glucosidase (PDB ID: 4J5T).

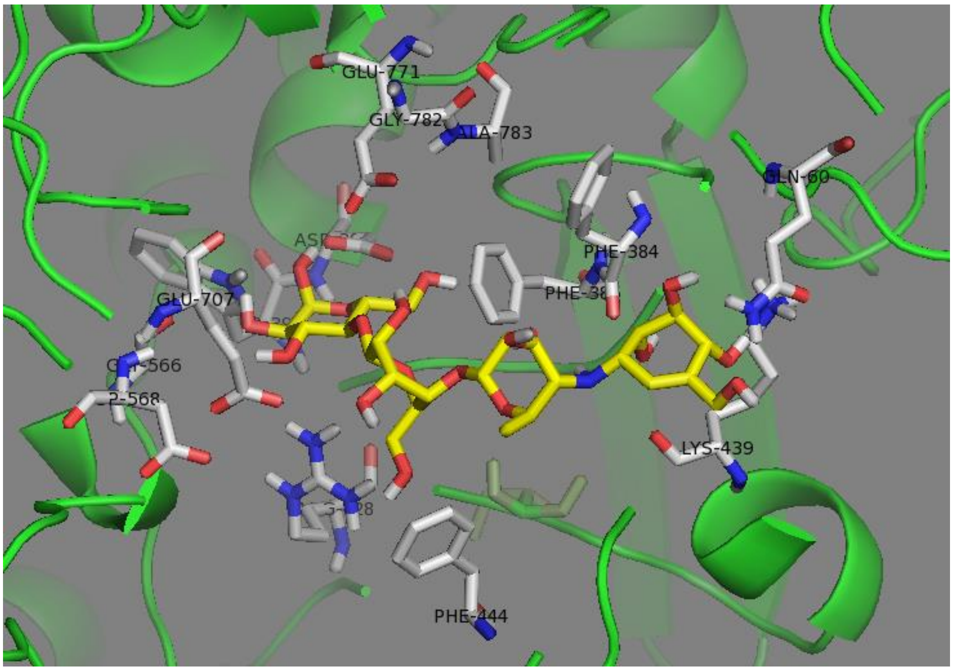

Figure S6: Three-dimensional representation of the docked pose of acarbose at the active site of α-glucosidase (PDB ID: 4J5T). The color coding for atoms is as follows: oxygen (O) is depicted in red, nitrogen (N) in blue, hydrogen (H) in white, carbon (C) in gray for amino acid residues, and yellow for the ligand. The catalytic pocket features several key residues essential for substrate binding and catalysis, including glutamic acid (GLU771), aspartic acid (ASP568, ASP392), arginine (ARG428), phenylalanine (PHE384, PHE385), tyrosine (TYR709), tryptophan (TRP391), valine (VAL446), isoleucine (ILE364), and glutamine (GLN442, GLN445), along with glutamic acid (GLU443), lysine (LYS439) and glycine (GLY566, GLY383).

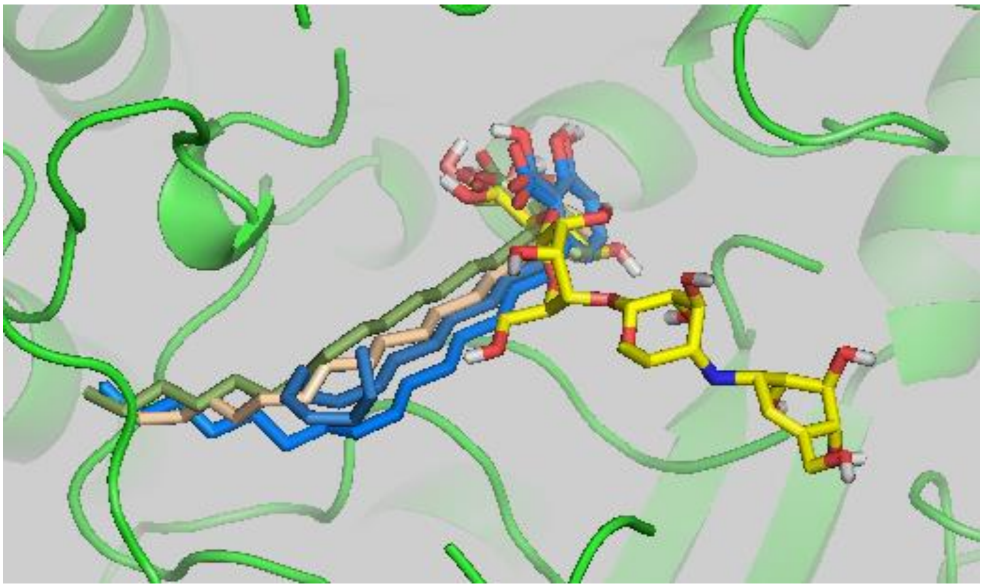

Figure S7: 3D visualization of the re-docking results demonstrating the superposition of the binding modes of AAn0, AAn1, AAn2, and AAn3, together with the reference inhibitor acarbose (with carbon in yellow), all occupying the same cavity in the α-glucosidase structure (PDB ID: 4J5T). The color coding for atoms is as follows: oxygen (O) is represented in red, nitrogen (N) in blue, hydrogen (H) in white for all. Carbon (C) in gray for amino acid residues, and carbon in blue, cyan, beige, and dark green for the ligands AAn0, AAn1, AAn2, and AAn3, respectively.

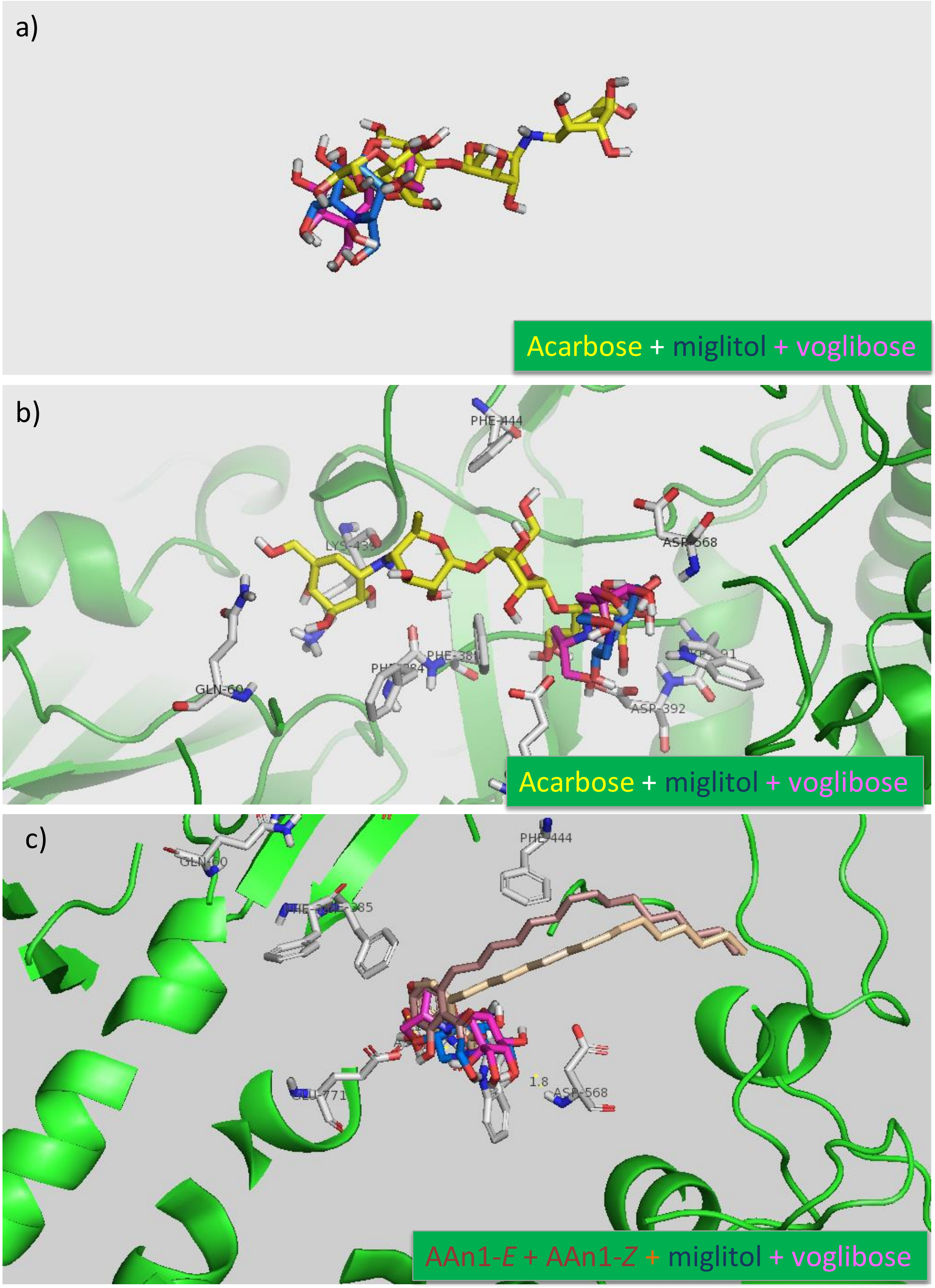

Figure S8: (a) 3D visualization showing the partial superimposition of the antidiabetic drugs voglibose (dark pink), miglitol (yellow), and the reference inhibitor acarbose (yellow) with RMSD = 0.00 Å, used as the standard in both in silico and in vitro assays (without showing the enzyme - PDB ID: 4J5T). (b) Enzyme-ligand complex showing all drugs binding to the same α-glucosidase pocket (with the enzyme shown - PDB ID: 4J5T). (c) 3D view showing miglitol, voglibose, and anacardic acids binding to the same pocket, forming hydrogen bonds with ASP568, ASP392, and TRP391. All compounds bind to the catalytic pocket.

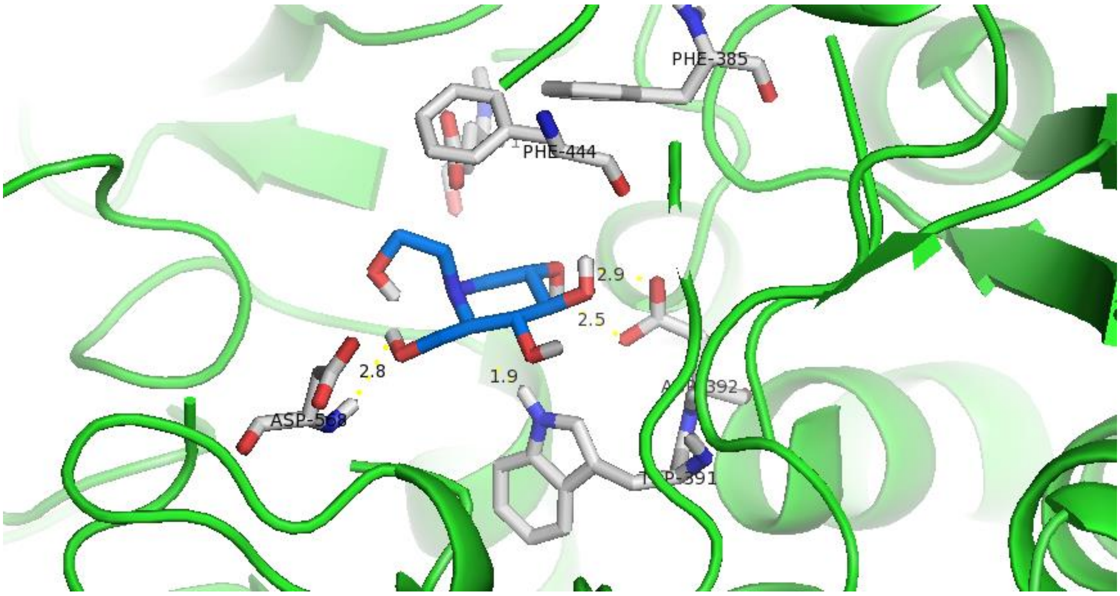

Figure S9: Three-dimensional representation of the binding interactions between miglitol (with carbon in blue in the center) and the amino acid residues at the active site of α-glucosidase (PDB ID: 4J5T). In yellow dashed line highlight the hydrogen bonds with the residues ASP568, ASP392, and TRP391.

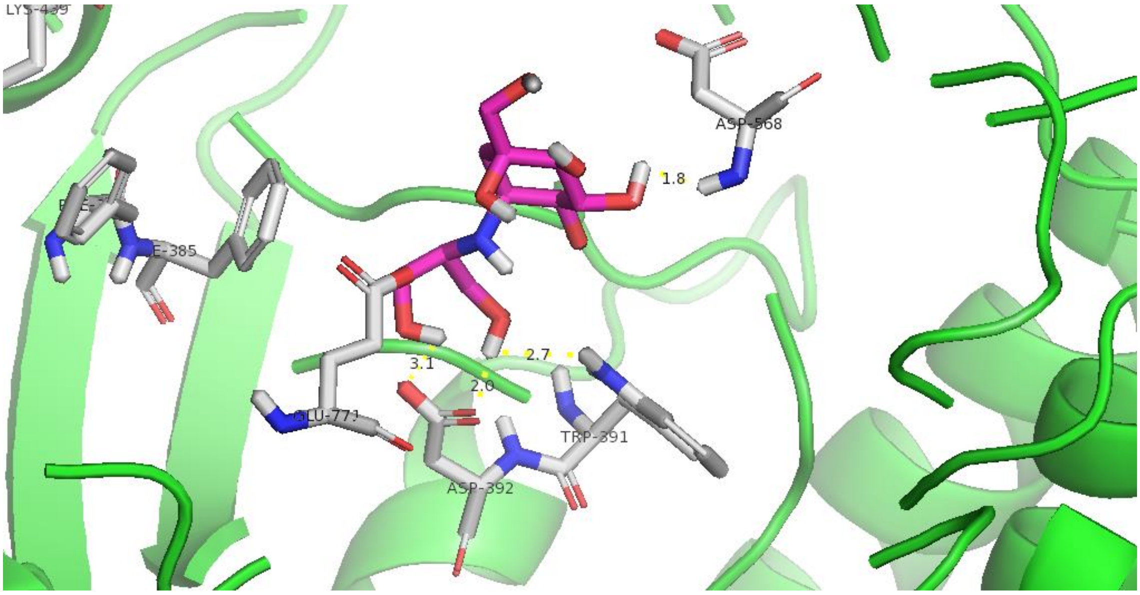

Figure S10: Three-dimensional representation of the binding interactions between voglibose (with carbon in yellow in the center) and the amino acid residues at the active site of α-glucosidase (PDB ID: 4J5T). In yellow dashed line highlight the hydrogen bonds with the residues ASP568, ASP392, and TRP391.

Experimental parameters and 2D/1D NMR spectra

Parameter	*Value*
Title	PROTON
Author	Gisele/Priscila
Sample	AAn1
Origin	Varian
Instrument	vnmrs
Solvent	DMSO-d_6
Temperature	26.0 °C
Pulse Sequence	s2pul
Experiment	1D
Probe	One_NMR_W023
Number of Scans	8
Receiver Gain	30
Relaxation Delay	10.000
Pulse Width	41.000
Acquisition Time	17.039
Spectrometer Frequency	599.57
Spectral Width	9615.4
Lowest Frequency	-1210.4
Nucleus	^{1}H
Acquired Size	16384

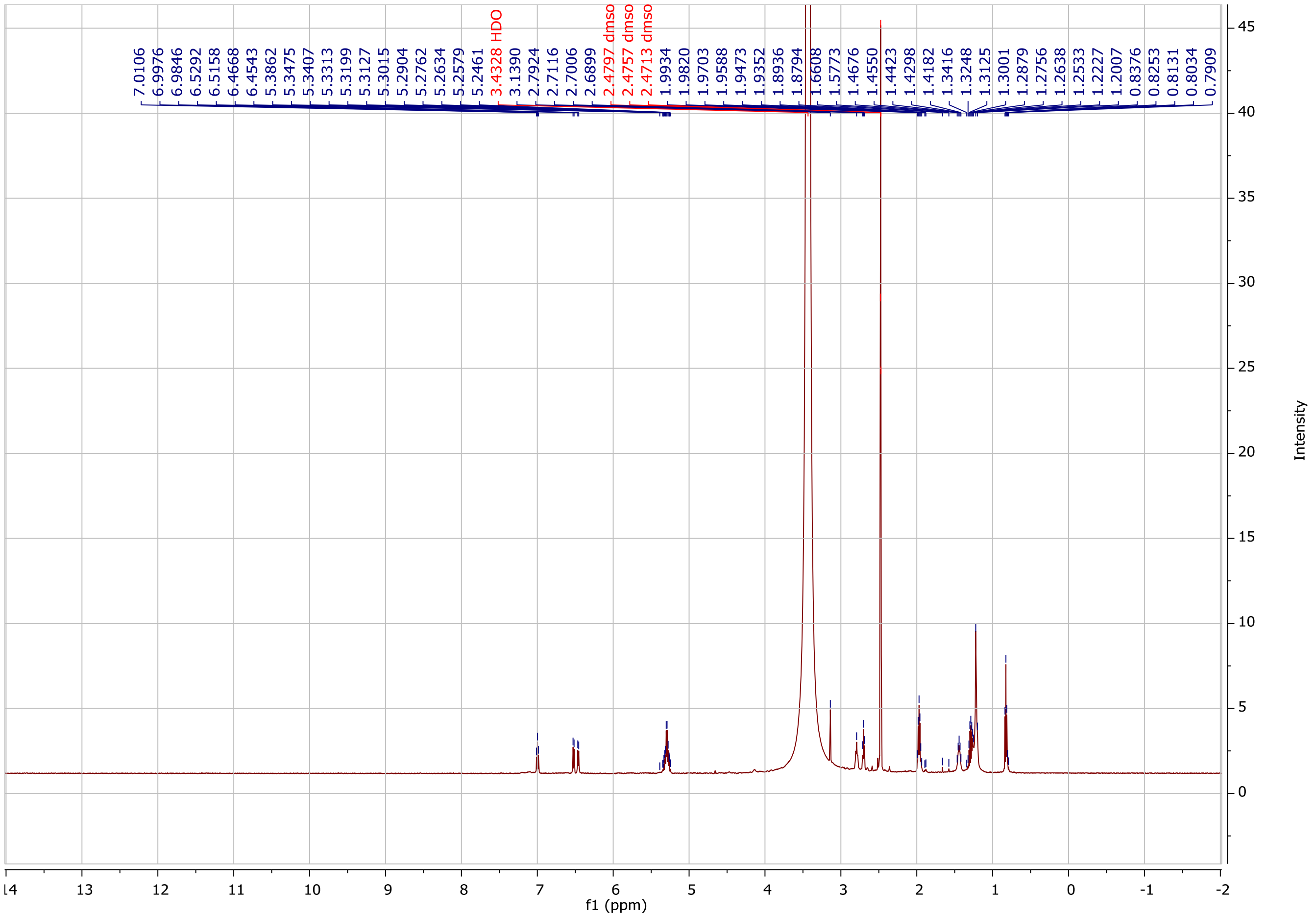

Figure S11. The ^{1}H NMR spectrum (600 MHz, DMSO-d_6) of AAn1.

Parameter	*Value*
Title	PRESAT
Author	Gisele/Priscila
Sample	AAn2
Origin	Varian
Instrument	vnmrs
Solvent	DMSO-d_6
Temperature	26.0 °C
Pulse Sequence	PRESAT
Experiment	1D
Probe	One_NMR_W023
Number of Scans	64
Receiver Gain	30
Relaxation Delay	20.000
Pulse Width	82.000
Presaturation Frequency	348.808
Acquisition Time	17.039
Spectrometer Frequency	599.57
Spectral Width	9615.4
Lowest Frequency	-1206.5
Nucleus	^{1}H
Acquired Size	16384
Spectral Size	65536

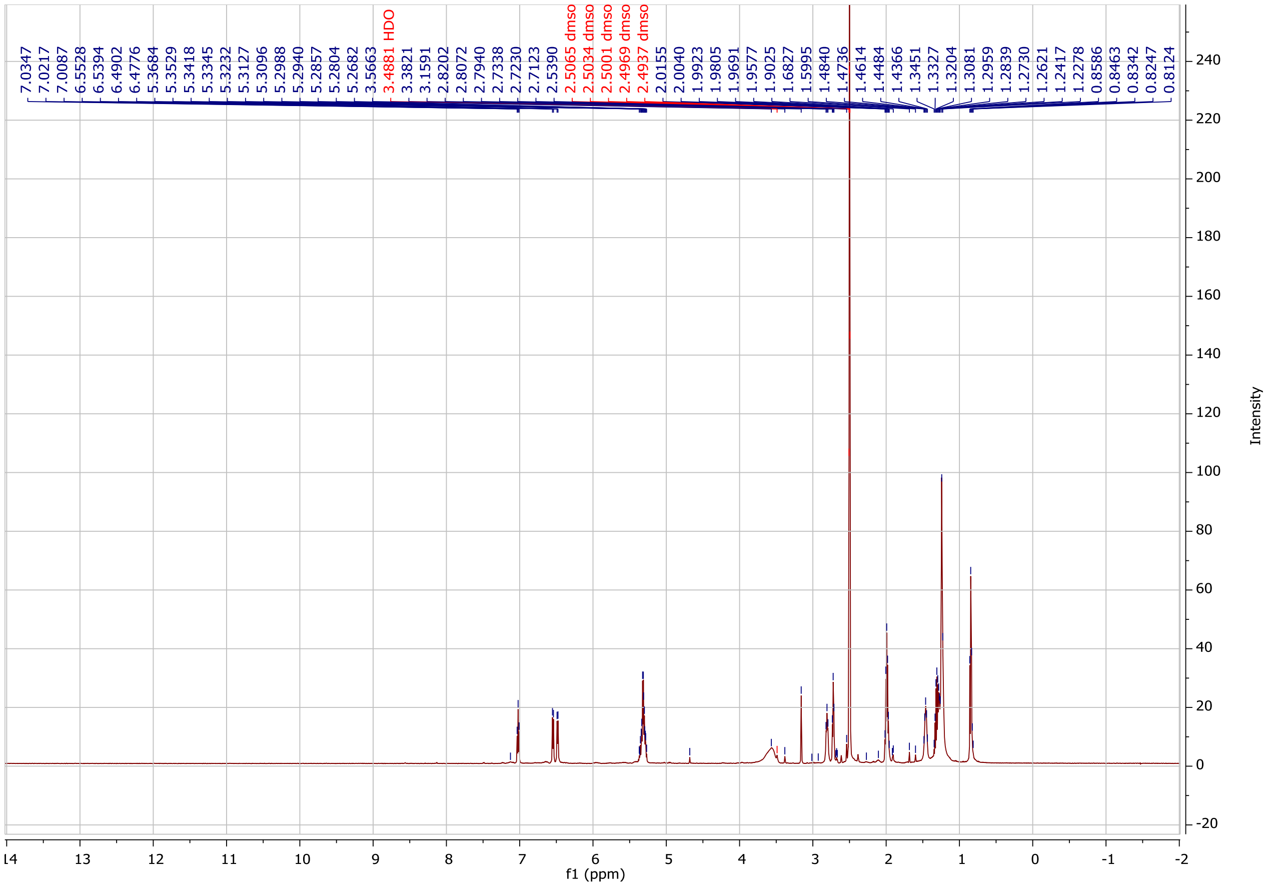

Figure S12. The ^{1}H NMR spectrum (600 MHz, DMSO-d_6) of AAn2.

Parameter	*Value*
Title	PRESAT
Author	Gisele/Priscila
Sample	AAn3
Origin	Varian
Instrument	vnmrs
Solvent	DMSO-d_6
Temperature	26.0 °C
Pulse Sequence	PRESAT
Experiment	1D
Probe	One_NMR_W023
Number of Scans	64
Receiver Gain	30
Relaxation Delay	20.000
Pulse Width	82.000
Presaturation Frequency	348.808
Acquisition Time	17.039
Spectrometer Frequency	599.57
Spectral Width	9615.4
Lowest Frequency	-1206.5
Nucleus	^1H
Acquired Size	16384
Spectral Size	65536

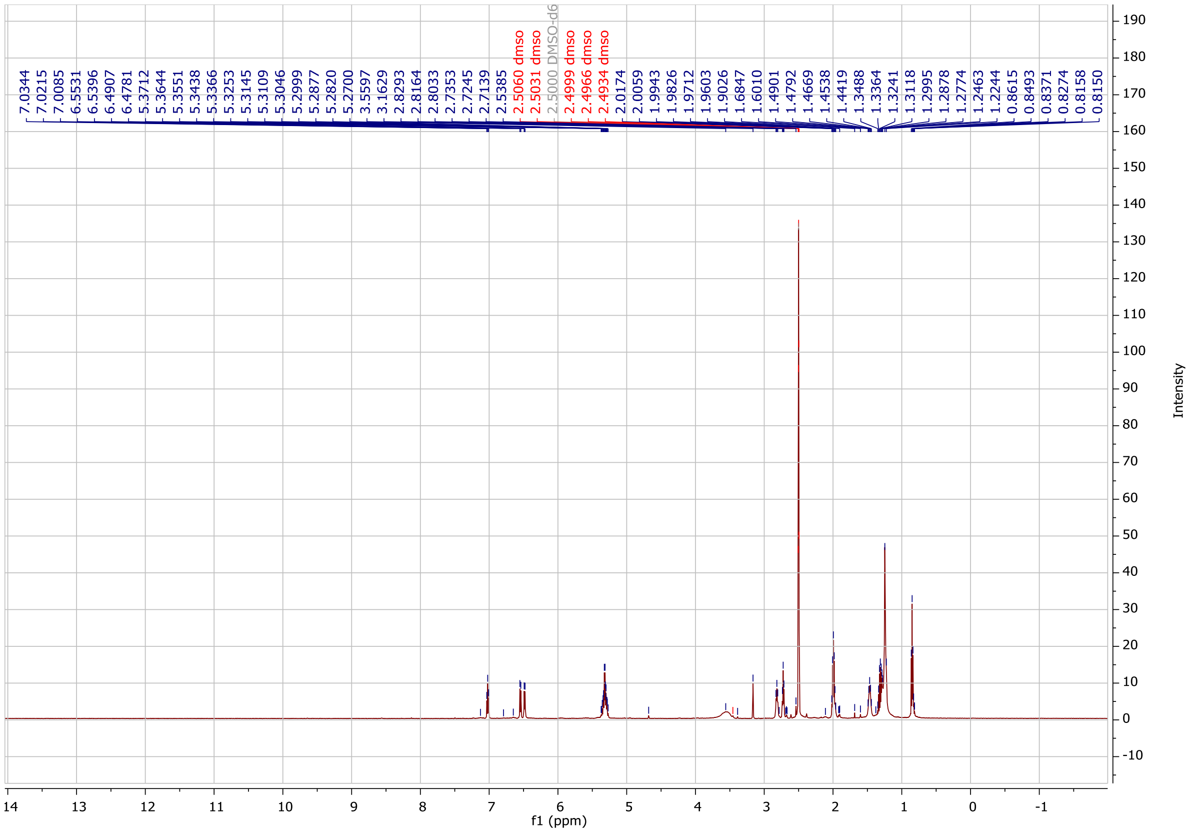

Figure S13. The ^1H NMR spectrum (600 MHz, DMSO-d_6) of AAn3.

Parameter	*Value*
Title	CARBON
Sample	AAn2
Origin	Varian
Instrument	vnmrs
Author	Gisele/Priscila
Solvent	DMSO-d_6
Temperature	26.0 °C
Pulse Sequence	s2pul
Experiment	1D
Probe	One_NMR_W023
Number of Scans	12000
Receiver Gain	30
Relaxation Delay	10.000
Pulse Width	41.937
Acquisition Time	0.8651
Spectrometer Frequency	150.78
Spectral Width	37878.8
Lowest Frequency	-2407.3
Nucleus	^{13}C
Acquired Size	32768
Spectral Size	65536

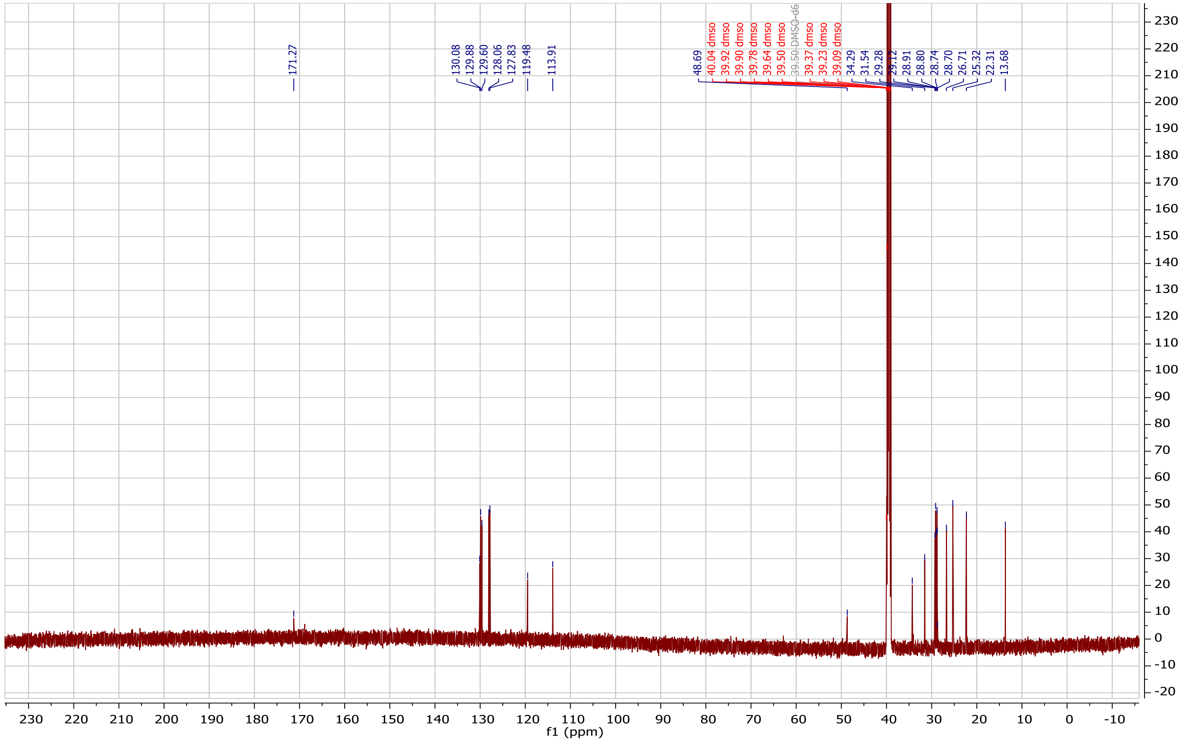

Figure S14. The ^{1}H NMR spectrum (150 MHz, DMSO-d_6) of AAn2.

Parameter	*Value*
Title	HSQC
Sample	AAn1
Origin	Varian
Instrument	vnmrs
Author	Gisele/Priscila
Solvent	DMSO-d_6
Temperature	26.0 °C
Pulse Sequence	HSQCAD
Experiment	HSQC-EDITED
Probe	One_NMR_W023
Number of Scans	64
Receiver Gain	28
Relaxation Delay	10.000
Pulse Width	84.000
Presaturation Frequency	345.911
Acquisition Time	0.2130
Spectrometer Frequency	(599.57, 150.77)
Spectral Width	(9615.4, 30154.5)
Lowest Frequency	(-1194.4, -1585.9)
Nucleus	(1H, 13C)
Spectral Size	(2048, 2048)
Acquired Size	(2048, 256)

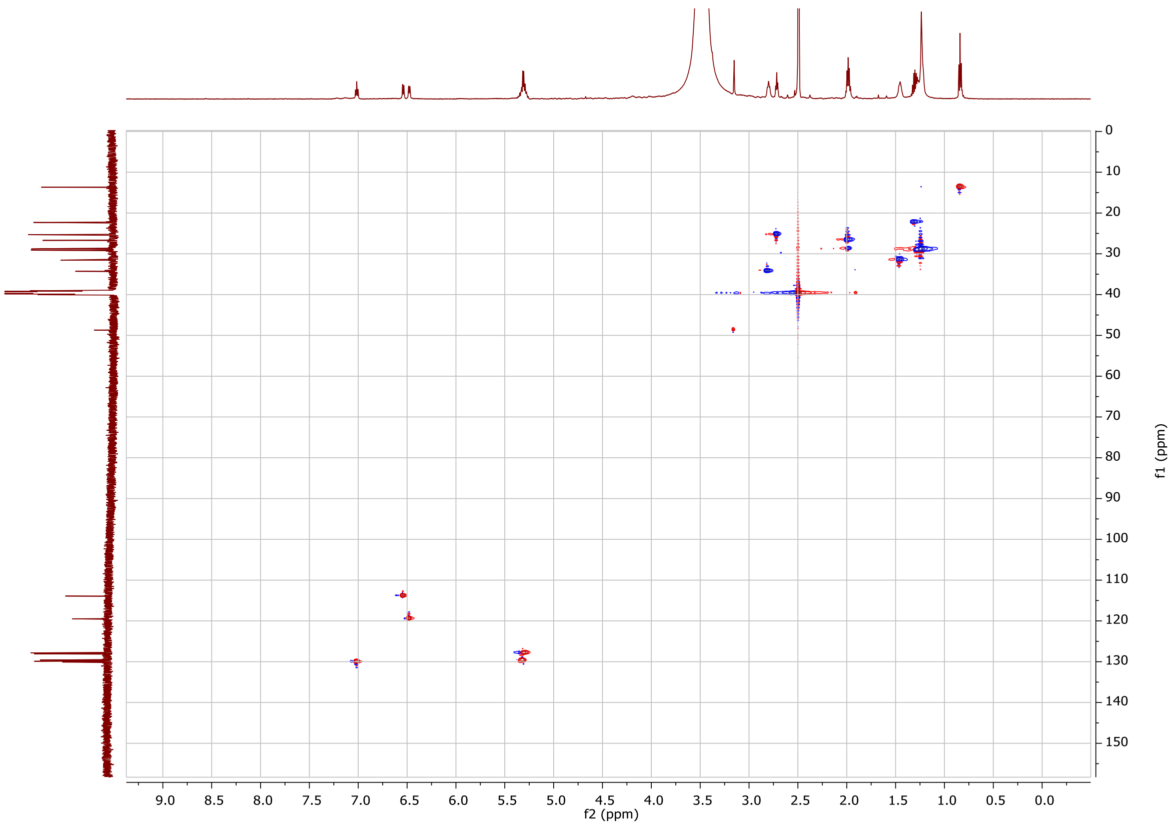

Figure S15. ^1H-^{13}C HSQC contour maps (600/150 MHz, DMSO-d_6) of AAn1.

Parameter	*Value*
Title	HSQC
Sample	AAn2
Origin	Varian
Instrument	vnmrs
Author	Gisele/Priscila
Solvent	DMSO-d_6
Temperature	26.0 °C
Pulse Sequence	HSQCAD
Experiment	HSQC-EDITED
Probe	One_NMR_W023
Number of Scans	64
Receiver Gain	28
Relaxation Delay	10.000
Pulse Width	84.000
Presaturation Frequency	345.911
Acquisition Time	0.2130
Spectrometer Frequency	(599.57, 150.77)
Spectral Width	(9615.4, 30154.5)
Lowest Frequency	(-1194.4, -1585.9)
Nucleus	(1H, 13C)
Spectral Size	(2048, 2048)
Acquired Size	(2048, 256)

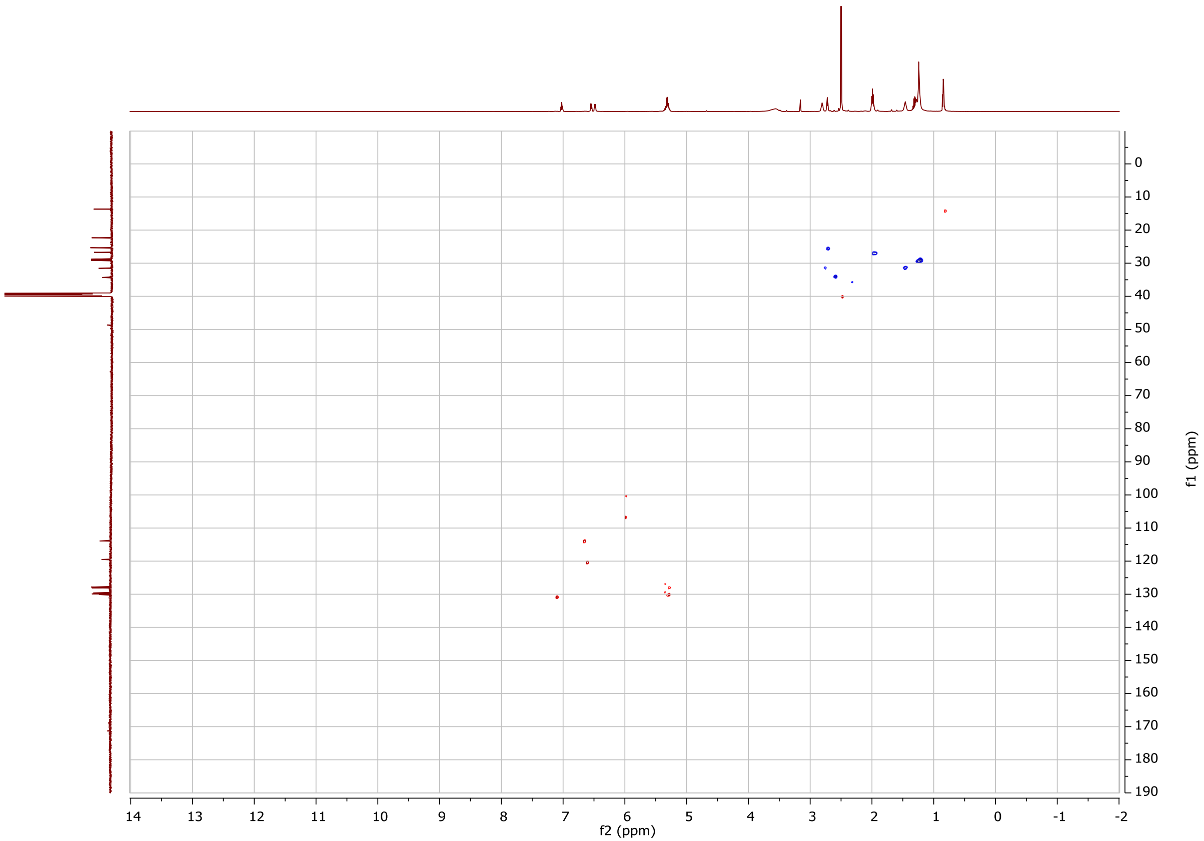

Figure S16. ^{1}H-^{13}C HSQC contour maps (600/150 MHz, DMSO-d_6) of AAn2.

Parameter	Value
Sample	HSQC
Comment	AAn3
Origin	Varian
Instrument	vnmrs
Author	Gisele/Priscila
Solvent	dmso
Temperature	26.0
Pulse Sequence	gHSQCAD
Experiment	HSQC-EDITED
Probe	One_NMR_W023
Number of Scans	128
Receiver Gain	28
Relaxation Delay	10.000
Pulse Width	82.000
Presaturation Frequency	362.134
Acquisition Time	0.1500
Spectrometer Frequency	(599.57, 150.77)
Spectral Width	(9615.4, 30154.5)
Lowest Frequency	(-1194.8, -1590.4)
Nucleus	(1H, 13C)
Acquired Size	(1442, 200)
Spectral Size	(2048, 2048)

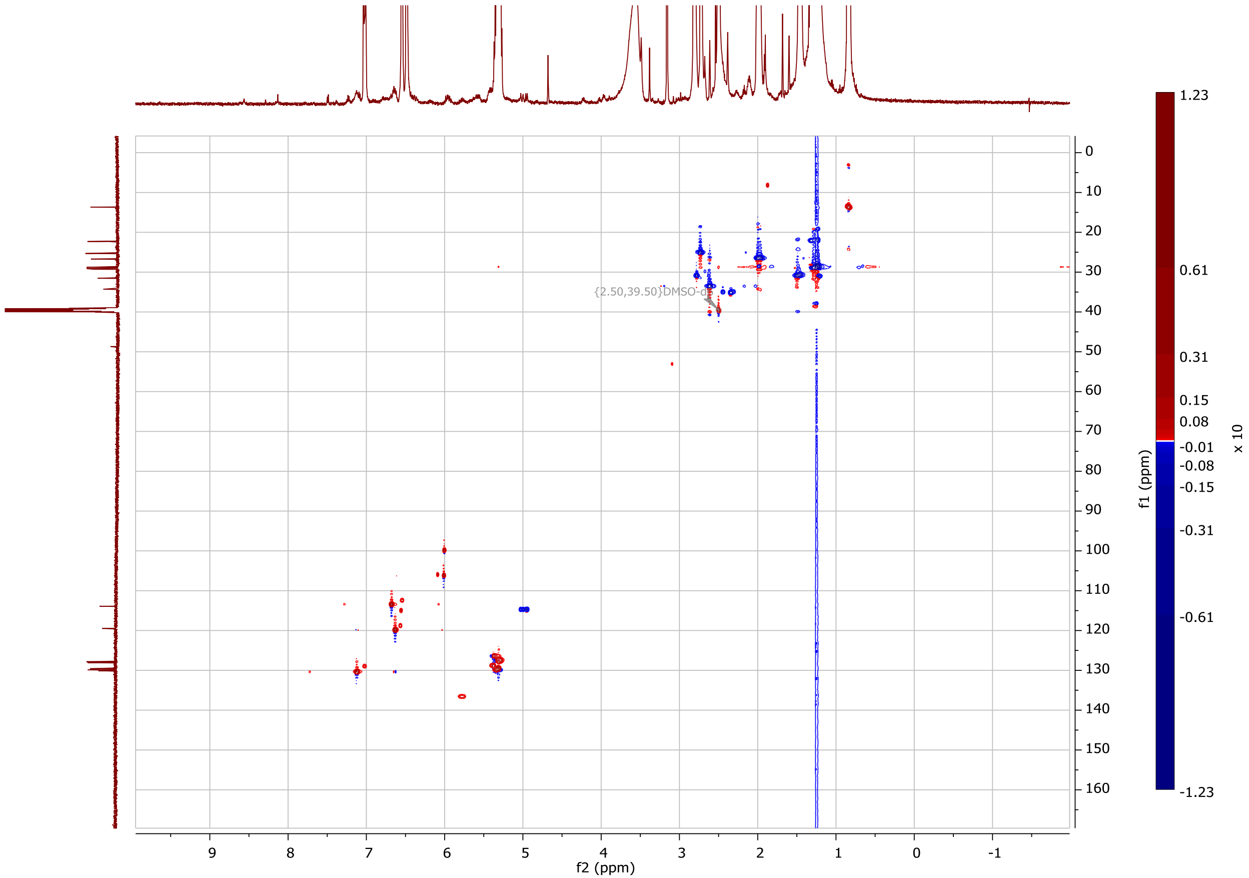

Figure S17. ^{1}H-^{13}C HSQC contour maps (600/150 MHz, DMSO-d_6) of AAn3.

Parameter	Value
Title	HMBC
Comment	AAn2
Origin	Varian
Instrument	vnmrs
Author	Gisele/Priscila
Solvent	DMSO-d_6
Temperature	26.0 °C
Pulse Sequence	gHMBCAD
Experiment	HMBC
Probe	One_NMR_W023
Number of Scans	64
Receiver Gain	28
Relaxation Delay	10.000
Pulse Width	84.000
Presaturation Frequency	345.732
Acquisition Time	0.2130
Spectrometer Frequency	(599.57, 150.78)
Spectral Width	(9615.4, 36182.7)
Lowest Frequency	(-1195.5, -2315.1)
Nucleus	(1H, 13C)
Acquired Size	(2048, 256)
Spectral Size	(2048, 2048)

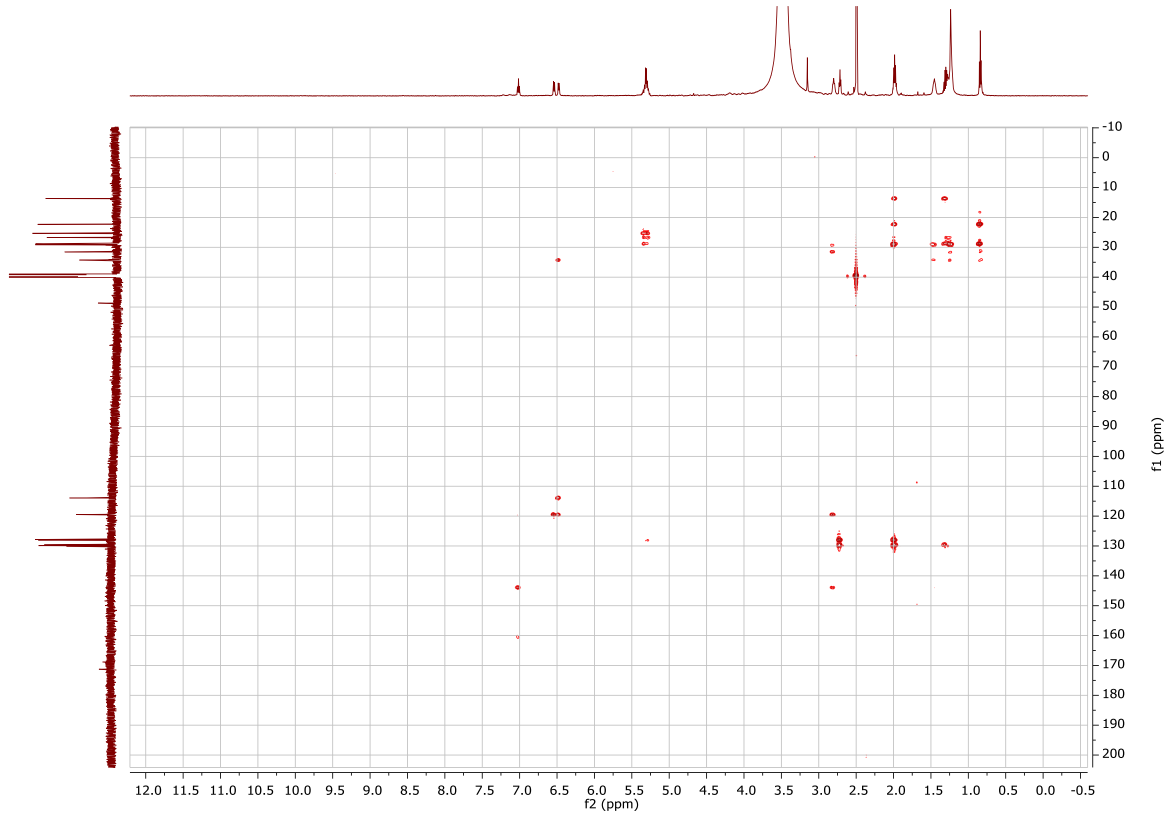

Figure S18. ^{1}H-^{13}C HMBC contour maps (600/150 MHz, DMSO-d_6) of AAn2.

Parameter	Value
Title	gCOSY
Sample	AAn2
Origin	Varian
Instrument	vnmrs
Author	Gisele/Priscila
Solvent	DMSO-d_6
Temperature	26.0 °C
Pulse Sequence	gCOSY
Experiment	COSY
Probe	One_NMR_W023
Number of Scans	128
Receiver Gain	28
Relaxation Delay	10.000
Pulse Width	82.000
Presaturation Frequency	362.134
Acquisition Time	0.1500
Spectrometer Frequency	(599.57, 599.57)
Spectral Width	(9615.4, 9615.4)
Lowest Frequency	(-1210.3, -1210.3)
Nucleus	(1H, 1H)
Acquired Size	(1442, 128)
Spectral Size	(2048, 2048)

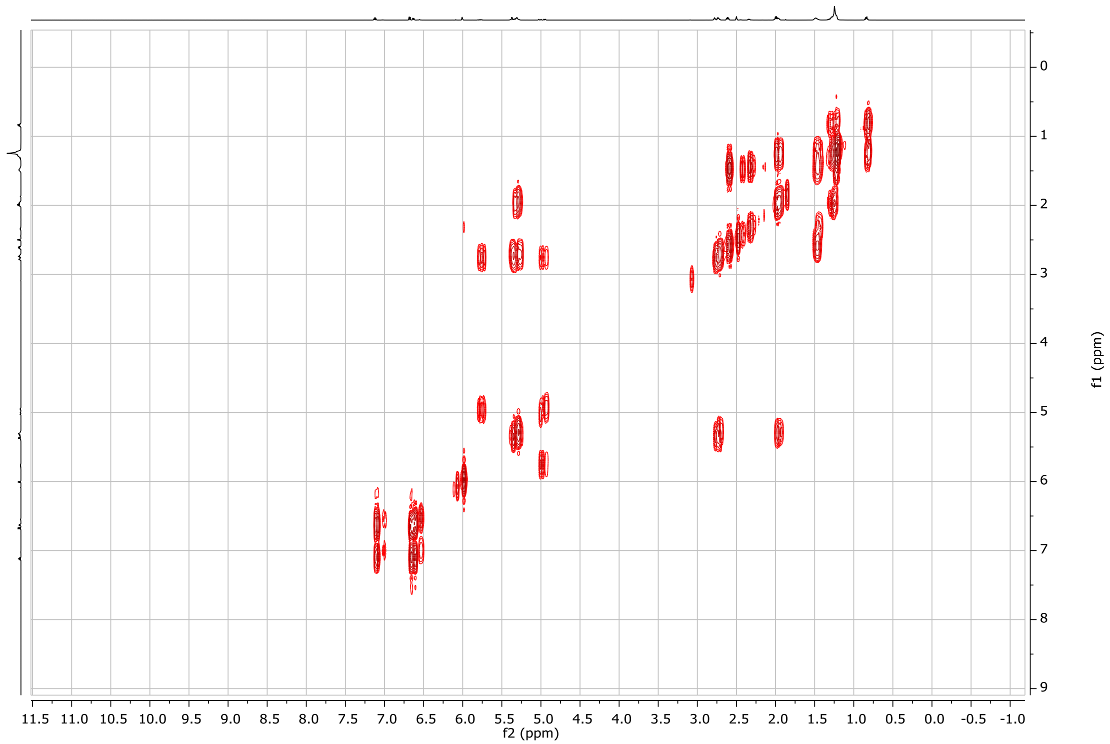

Figure S19. ^{1}H-^{13}C COSY contour maps (150/150 MHz, DMSO-d_6) of AAn2.
